# Pretraining for Large-Scale Functional Connectome Fingerprinting Supports Generalization and Transfer Learning in Functional Neuroimaging

**DOI:** 10.1523/ENEURO.0370-25.2026

**Published:** 2026-08-21

**Authors:** Mattson Ogg, Lindsey Kitchell

**Affiliations:** Research and Exploratory Development Department, Johns Hopkins Applied Physics Laboratory, Laurel, Maryland 20723

**Keywords:** deep learning, fMRI, functional connectome, transfer learning

## Abstract

Functional MRI (fMRI) currently supports a limited application space stemming from modest dataset sizes, large interindividual variability, and heterogeneity among scanning protocols. These constraints have made it difficult for fMRI researchers to take full advantage of modern deep-learning tools that have revolutionized other fields such as NLP, speech transcription, and image recognition. To help address these issues, we scaled up functional connectome fingerprinting as a neural network pretraining task, drawing inspiration from speaker recognition research, to learn a generalizable representation of brain function. This approach achieves strong performance for neural fingerprinting on a previously unseen scale, across multiple public fMRI datasets (individual recognition from held-out scan sessions, 93% on MPI-Leipzig, 94% on NKI-Rockland, 73% on OASIS-3, and 99% on HCP). Performance is maintained even when evaluation scan duration is truncated to <2 min. We show that this representation can also generalize to support accurate neural fingerprinting for completely new datasets and participants of either sex not used in training. Finally, we demonstrate that the representation learned by the network encodes features related to individual variability that partially transfers to new tasks. These results support the development of scalable transfer-learning approaches for future clinical and cognitive neuroimaging applications.

## Significance Statement

Deep-learning models that leverage the increasing scale of available functional MRI data could address fundamental generalization challenges. We drew inspiration from other domains that have successfully used deep learning to address these problems, namely, human language technology, to guide our approach for addressing these challenges in neuroimaging. Our pretraining method achieves strong performance for functional connectome fingerprinting, achieving very high recognition accuracy across different tasks, scanning sessions, and acquisition parameters, even when the duration of a scan is limited to <2 min. Representations learned by our model could be repurposed to recognize new individuals from new datasets and to predict new participants' cognitive performance and traits.

## Introduction

Functional MRI (fMRI) has delivered incredible scientific insights into cognition (Haxby et al., 2001; Padmala and Pessoa, 2008; Bonnici et al., 2012; Hsu et al., 2017; Pereira et al., 2018), but it has been difficult to find applications for fMRI beyond research (Ramduny and Kelly, 2025). Generalizing models and algorithms beyond a particular dataset or scanner is often difficult (Dufumier et al., 2022; Wang et al., 2022). Two critical constraints contribute to these limitations: (1) nontrivial interindividual variability in brain function (Gratton et al., 2018) and (2) small sample sizes (i.e., tens of participants), given the cost of acquiring fMRI data (Szucs and Ioannidis, 2020). These constraints compound one another: limited sample sizes reduce one's power to map the key dimensions of interindividual variability that exist in the larger population (Marek et al., 2022).

Deep learning and large data volumes have allowed computer vision, language, and speech researchers to build models that learn the structure of these domains (LeCun et al., 2015) such that they can now support high off-the-shelf performance for downstream users and practitioners. Importantly, after training, these models can be repurposed or fine-tuned, where features learned from large-scale training data support high performance on new tasks even when fine-tuning data is limited (a process called transfer learning; Huh et al., 2016; Krishnan et al., 2022). This approach revolutionized these research spaces and transformed how image, audio, and text data is used. For example, in speaker recognition, neural networks are first trained on large quantities of data to achieve high accuracy discerning individuals (Nagrani et al., 2017; Snyder et al., 2018). These models can then be repurposed to address new tasks, including classifying clinical states like Alzheimer's (Pappagari et al., 2020) and Parkinson's disease (Moro-Velazquez et al., 2020) from speech.

Large-scale data collection efforts for MRI and data-sharing consortia (on the scale of thousands of participants) have been launched to overcome similar limitations in neuroimaging (Markiewicz et al., 2021). The increasing scale of neural data has supported different subfields of “neural fingerprinting” wherein high-level, individualized representations characterize the patterns of one's neural activity relative to the larger population (Wachinger et al., 2014; Kumar et al., 2017, 2018; note these are developed using a dataset's anonymized subject identifiers, not any real-world identities). Functional connectome fingerprinting in particular has a long history of research and has proven to be a powerful representation of brain function by correlating activity from different brain regions over time (Amico and Goñi, 2018; Wang et al., 2019; Abbas et al., 2020; Sarar et al., 2021; Van De Ville et al., 2021; Griffa et al., 2022). Functional connectome fingerprinting has achieved impressive accuracy for recognizing individuals across tasks (Finn et al., 2015) and can be used to predict cognitive performance (Rosenberg et al., 2016, 2020) and traits of individuals (Greene et al., 2018). Connectome fingerprints have also been suggested as a way to support personalized and reliable clinical diagnostics (Ramduny and Kelly, 2025). While impressive, most studies rely on limited datasets of hundreds of participants, far removed from the scale of individuals that may be required to accurately predict cognitive function and mental health variables (Marek et al., 2022; Spisak et al., 2023). Additionally, there has been very limited study of the generalization of functional connectome fingerprinting to recognizing individuals from new datasets or to perform new tasks, and deep learning is only starting to be applied in this area (Sarar et al., 2021).

Beyond neural fingerprinting, progress has been made in generalizing fMRI analytics across scanners and datasets (Gao et al., 2023; Horien et al., 2023; Yip et al., 2023), but it is not a solved problem. For example, a recent set of reports developed algorithmic solutions that find common trends among underlying phenotypes that are common across datasets (He et al., 2022; Chen et al., 2024), improving cross-dataset predictive accuracy. However, these approaches do not distill a generalizable representation that could help for the analysis or exploration of a new, smaller dataset. Some self-supervised foundation models have also been introduced for fMRI, but it remains to be seen if they quantify exactly the right kinds of features to support clinical utility and personalization. For example, recent work has adapted self-supervised pretraining methods to train foundation models for fMRI (Caro et al., 2023; d’Ascoli et al., 2025; Yang et al., 2025) that might serve a similar function for downstream applications where data are limited. These foundation models may prove to be powerful tools, but it is currently unclear how they will ultimately be scaled and deployed in the face of heterogeneous fMRI scanning hyperparameters. Specifically, many approaches predict hemodynamic time series, but differing TRs in downstream datasets would cause misalignment. We take a different approach, relying on the long, productive history of (supervised) connectome fingerprinting as a pretraining task, which may serve as a useful springboard for generalization as well as help support reliable and personalized clinical applications.

## Materials and Methods

fMRI is approaching a point where breakthroughs that combine deep learning and large datasets can be realized thanks to expanding database efforts (Markiewicz et al., 2021) and advances in standardized preprocessing tools (Esteban et al., 2019). To explore this, we pretrained neural network representations, which we call “brain2vec” for short, that would learn useful features from large, composite datasets for later use on entirely new data and tasks. We based our experiments on the demonstrated utility of functional connectome fingerprinting (Finn et al., 2015) and drew inspiration from work on speaker recognition (Nagrani et al., 2017; Snyder et al., 2018) and benchmarking for human language technologies (SUPERB; Yang et al., 2021) in developing this approach.

Functional connectome fingerprinting illuminates a core question in cognitive neuroscience: how can we quantify the variability that exists among individuals and their unique cognitive patterns? To meet this challenge, we looked to the task of speaker recognition, which has made progress on related problems in the speech domain, to guide our methodology [primarily Nagrani et al. (2017) but also Snyder et al. (2018) and to a lesser degree Chen et al. (2022)]. In speaker recognition, large-scale supervised pretraining has yielded generalizable and transferable representations. There are several similarities that make this comparison particularly useful: First, unique subject identifiers are standard in fMRI datasets, and these provide a straightforward target for supervised classification during pretraining (similar to speaker recognition). Second, forcing the model to distinguish high numbers of unique participants requires the model to learn nuanced features about individual variability in order to accurately distinguish participants. Therefore, the features that the model learns will likely be important in other datasets (similar to transfer learning of pretrained speaker recognition embeddings to “speaker verification” tasks for new individuals). Third, fMRI data from participants performing different tasks in the scanner increase pretraining difficulty and encourage generalizability by forcing the model to learn representations of individuals that are independent of a given pattern of task responses (similar to text-independent speaker recognition). Fourth, some robustness to particular scanners or recording conditions can be learned by training on data from a diverse set of scanning facilities (similar to training on data from different audio domains and datasets, e.g., phone conversations, reading, interviews). Fifth, the temporal nature of fMRI is not dissimilar from speech data: both time series can be subsampled to generate additional training examples and improve robustness for future samples where duration is limited. Finally, where multiple scanning sessions are present (e.g., from different functional scanning runs but ideally from separate scanning days), data can be completely held out to form a validation set (similar to held-out audio recordings).

### Data and preprocessing

We compiled pretraining [HCP1200, Van Essen et al. (2013); NKI-Rockland, Nooner et al. (2012); MPI-Leipzig Mind-Brain-Body, Babayan et al. (2019); Mendes et al. (2019); 1,000 Functional Connectomes, Biswal et al. (2010); AOMIC ID1000, Snoek et al. (2021); OASIS-3, LaMontagne et al. (2019)] and held-out transfer learning [AOMIC PIOP1 and PIOP2; LA5c, Poldrack et al. (2016); and data from Spreng et al. (2022); Setton et al. (2023); https://openneuro.org number ds003592] datasets from publicly available fMRI databases (https://db.humanconnectome.org for HCP, https://fcon_1000.projects.nitrc.org for NKI-Rockland and 1,000 Functional Connectomes, https://central.xnat.org for OASIS-3, and the rest were acquired from https://openneuro.org). We only retained individuals from OASIS-3 who had a Clinical Dementia Rating score of 0 for all scans, to maintain a training corpus of only healthy study participants, as all other studies included in the training set targeted healthy, nonclinical populations. We also only retained data from a subset of 21 sites in the 1,000 Functional Connectomes study which had complete data (both a functional scan and a structural scan; Table 1). We ignored any scans with fewer than 100 TRs. All data were minimally preprocessed to arrive at a version of the data in a common volumetric MNI152 space along with a set of confounds for later processing. We carried out minimal preprocessing in-house using fmriprep (version 20.2.6; Esteban et al., 2019) for all datasets, except for HCP, for which we used the preprocessed MNI152 derivatives released by the human connectome project (Glasser et al., 2013), and the AOMIC datasets (for which we used the publicly provided derivatives generated by fmriprep version 1.4.1). All fmriprep runs were executed with the “ignore slice timing” and “susceptibility distortion correction” flags. Text output generated by fmriprep describing these runs for a representative subject from each dataset is available in the supplemental material (Extended Data 1).

**Table 1.** Description of data

	Training						Held-out			
	HCP	NKI-Rockland	MPI-Leipzig	1000 Func. Conn.^a^	AOMIC ID1000	OASIS-3	AOMIC PIOP1	AOMIC PIOP2	LA5c	ds003592^b^
Age range (min., max.)	22, 36+	6, 85	20, 80	8, 74	19, 26	42, 95	18, 26	18, 25	21, 50	18, 89+
Gender (M, F %)	46, 54.4%	38.7, 60.2%	59.4, 40.6%	41.2, 53.4%	48, 52%	38.5, 61.5%	41.2, 55.6%	42.5, 57.1%	57, 43%	43.9, 55.8%
Unique participants	1098	1318	318	816	881	556	216	226	265	285
Number of scanning days per participant (min., max.)	1, 2	1, 5	1, 2	1, 1	1, 1	1, 6	1, 1	1, 1	1, 1	1, 1
Functional Tasks	EMOTION, GAMBLING, MOTOR, RELATIONAL, WM, LANGUAGE, REST, SOCIAL	Rest, BREATHHOLD, CHECKERBOARD	Rest	Rest	Movie watching	Rest	workingmemory, gstroop, restingstate, emomatching, faces, anticipation	workingmemory, stopsignal, restingstate, emomatching	task-rest, task-scap, task-bart, task-taskswitch, task-stopsignal, task-pamenc, task-pamret	Rest
Number of full scans	19134	11255	1016	816	881	2101	1236	895	1718	569

a Sites from the 1,000 Functional Connectomes dataset that were used: “AnnArbor_a,” “Baltimore,” “Bangor,” “Beijing_Zang,” “Berlin_Margulies,” “Cambridge_Buckner,” “Leiden_2200,” “Milwaukee_a,” “Milwaukee_b,” “Munchen,” “Newark,” “NewYork_a,” “NewYork_b,” “Queensland,” “Ontario,” “Oulu,” “Oxford,” “SaintLouis,” “PaloAlto,” “Taipei_a,” “Taipei_b.”

b Trait and cognitive performance tasks used: “vpa_imm_tot,” “vpa_delay,” “vpa_fr_delay,” “associative_recall,” “nihcog_rey,” “shipley_vocab,” “nihcog_orr,” “nihcog_pva,” “nihcog_psm,” “nihcog_lswm,” “nihcog_dccs,” “nihcog_flanker,” “trails_b_a,” “sdmt_oral,” “nihcog_crystallizedcomp,” “nihcog_fluidcomp,” “nihcog_totalcomp,” “episodic_index,” “semantic_index,” “executive_index,” “bfas_openness,” “bfas_conscientiousness,” “bfas_extraversion,” “bfas_agreeableness,” “bfas_neuroticism,” “mmse.”

After minimal preprocessing, all data underwent additional processing via nilearn in python (version 0.8.1). Data were parcellated into a 100-node Schaefer atlas (Schaefer et al., 2018; based on the 17-network atlas; Yeo et al., 2011), standardized (*z*-scored), detrended, and low-pass filtered at 0.08 Hz (this is the only filter applied, similar to prior work; Yeo et al., 2011). We then removed confounds for 12 motion parameters, CSF, white matter, and global signal and then dropped the first five samples in the time series. The fMRI time series was then used to derive connectivity matrices (made up of either standard or partial correlation values) for the full scan. The model takes these matrices made up of correlation *r* values as input. For generating additional examples via temporal subsampling, the time series was also subdivided into 100 s segments (with 50% overlap) before deriving additional connectivity matrices. We ignored any data that were not successfully processed by fmriprep or lacked a confounds file. Note that only motion derivatives were available for the HCP data, so preprocessing HCP did not involve CSF or white matter derivatives. A follow-up experiment confirmed that preprocessing the entire training corpus removing only motion derivatives from the time series that were available in HCP (i.e., no CSF or white matter removal) had very limited impact on our results. The control experiment resulted in nearly identical pretraining (validation) performance when all the data were cleaned only with motion parameters. We note that the 100 s temporal subsampling window we used is a very limited duration but aligns with suggested lower bounds for fMRI analyses that aim to localize activity in time (which is a stricter temporal requirement than ours; Leonardi and Van De Ville, 2015; Zalesky and Breakspear, 2015) and preserves individually identifiable information (Van De Ville et al., 2021).

To monitor neural fingerprinting performance during pretraining, we constructed a validation set by holding out one scanning session (i.e., one complete day of scanning) from every participant with more than one scan session. These held-out scans were separated from the other scans by days (in the case of HCP) or years (in the case of NKI-Rockland or OASIS-3). The held-out scan was always the last scan for each participant, with the exception of the MPI data. For MPI, the second session, “ses-02” (their Neuroanatomy & Connectivity Protocol), contained significantly more functional data than the first session, so we chose the second session for training, and the first session, “ses-01,” was used for validation. Note that partitioning the validation data this way means the HCP portion of the validation data involves many tasks not seen during training, since different tasks were done in the scanner across days. The validation set always involved the full-duration scans, ignoring the subsampled scans, except for follow-up analyses where we investigated model accuracy using the connectivity matrices corresponding to the first 100 s time window. Note that some participants (from NKI-Rockland or OASIS-3) or datasets (1,000 Functional Connectomes, AOMIC ID1000) had only one scan. Therefore, these participants only appear in the training dataset and were not used for validation.

### Model architecture and pretraining

**Figure 1.**
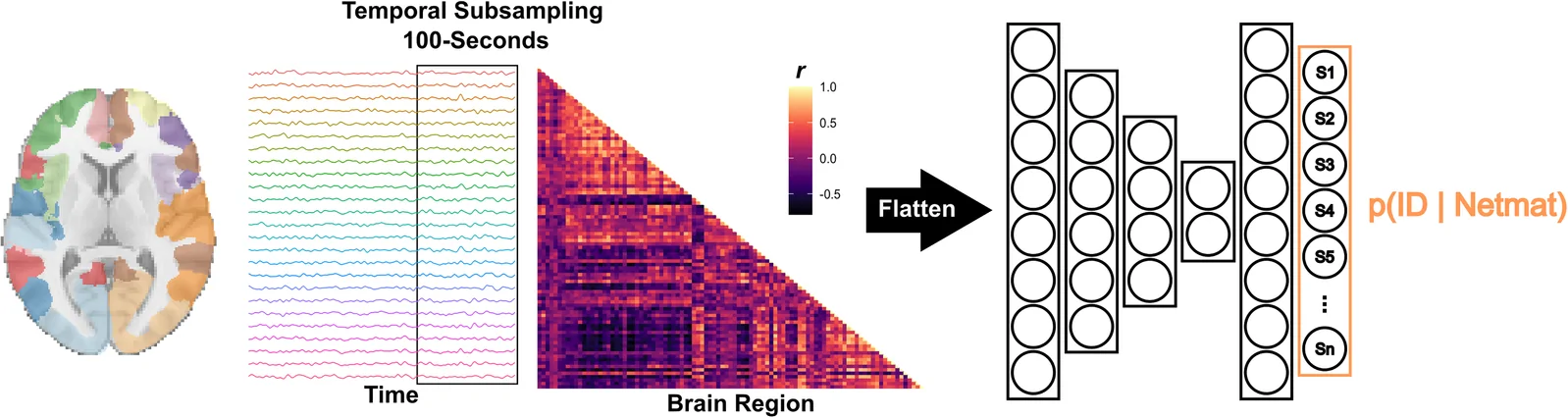
Schematic of the subject classification pretraining pipeline. fMRI data are parcellated into a 100-node atlas, and the time series is subsampled, converted into a network connectivity matrix and fed into the neural network. Here we show a standard correlation netmat and a deep “four-layer” (plus embedding layer) neural network configuration. The network's objective is to identify the participant ID with which the network connectivity matrix was associated.

We first trained feedforward neural network models to identify individuals in the training dataset from network connectivity matrices derived from their fMRI scans. We explored two model architectures. The “shallow” or “one-layer” model architecture comprised a single linear block (made up of a linear layer, leaky-ReLU activation, batch-norm, and dropout) followed by a linear output layer with one activation unit for each participant (4,987 units). The “deep” or “four-layer” model architecture comprised four linear blocks followed by a linear embedding layer (made up of a linear layer, leaky-ReLU activation, and batch-norm) and then a linear output layer (Fig. 1). The single linear block in the shallow model doubled as the embedding layer, with the embedding taken before the dropout layer. The input layer of the deep network was four times the size of the embedding layer with the number of activation units in the deep model halved with every subsequent linear block until the embedding layer in order to encourage the model to distill a more abstract representation at the deeper layers. This also kept the dimensionality of the embeddings from the deep model and the shallow models consistent. All linear layers were initialized with a Kaiming normal function; batch-norm layers had a momentum of 0.1 and did not have learnable affine parameters. Leaky-ReLU layers had operations executed in-place. We used pytorch (version 1.9.1) and pytorch-lightning (version 1.5.10) for neural network training and fine-tuning.

Models were trained for 50 epochs to minimize cross-entropy loss with a learning rate of 0.0001 using an Adam optimizer (weight decay, 0.0001) and a batch size of 64. If there was no improvement in validation loss for three epochs, the learning rate was reduced. Between different numbers of functional sessions and different scan durations, participants had different numbers of training examples. Thus, we applied a class weighting in our loss function to help normalize this. Participants (i.e., the target classes) with fewer training examples received a higher weight, and participants with more training examples had a reduced weight. We report accuracy along with macroaveraged precision, recall, and F1 scores. We evaluated above-chance performance (relative to the number of unique individuals in each validation set) using binomial tests, Bonferroni corrected for the number of models we trained (50), and the number of validation tests we ran (five total, overall performance as well as performance for each of four validation datasets). We used binomial tests to assess statistical significance and above-chance performance because many analyses involved direct evaluation of classification accuracy relative to analytically defined chance levels across a large number of model and dataset combinations. Our use of Bonferroni’s corrections for multiple comparisons served to maintain additional conservative statistical control.

Training runs encompassed a sweep across different model configurations and hyperparameters. We examined the effect of using different correlations in the input connectivity matrices (standard or partial correlations), model depth (shallow or deep model architectures), model size (embeddings of 1,024, 512, 256, 128 units), and dropout (0, 25, or 50%). Models were compared based on their performance after 50 epochs.

We provide scripts to run inference for minimally preprocessed data for the best-performing models that we describe and analyze below (i.e., to process and parcellate data to generate input connectivity matrices and to generate embeddings; see Data Availability section and Extended Data 2 for further details).

#### Ablation analyses

In order to understand which brain regions were most essential for the model's representation of individual variability, we removed (ablated) portions of the input connectivity matrix and recorded any corresponding changes in validation accuracy. The ablation analysis zeroed out the rows and columns of the connectivity matrices that corresponded to regions within each of the 17 Yeo networks before presenting them to the model for inference [drawing inspiration from the saliency map analysis run by Lee et al. (2022)]. For each of the 17 network ablations, we recorded validation accuracy and compared this with performance on the unablated data to obtain a delta for each ablation. Along with reporting changes in accuracy, these differences were then normalized between 0 and 1 by dividing by the delta for the network with the highest difference between ablated and unablated performance. We evaluated the statistical significance of each ablated model using a binomial exact test on the discordant paired outcomes of each ablated model relative to the original model. Note we focus on networks because the models were generally robust to zeroing out individual nodes (brain regions), thus producing very little change in performance, but nodewise ablation analyses are included in Extended Data Figures 4-1 and 4-2 for completeness. Finally, we report cumulative ablation analyses, where instead of ablating individual networks, networks were iteratively added to the ablation set in order of how much they reduced model accuracy in the first analysis.

#### Baseline comparison approaches

We used two baseline approaches to understand how our model's performance fared relative to standard methods (which we describe as the “correlation baseline method” below) and to understand the usefulness of our neural network's intermediate representation layers (which we describe as the “fully connected baseline method” below). Baseline methods were applied to all the features that were input to our models (100-node input connectivity matrices made up of standard or partial correlations). Our correlation baseline method was an implementation of the method described by Finn et al. (2015). This approach recognizes individuals by correlating connectivity matrices in the validation set to all other connectivity matrices in the training data. If the highest correlation was with a scan from the same participant, this was marked as a correct identification. Our implementation of this approach was carried out within dataset for computational efficiency (many fewer *n*-by-*n* comparisons), but we note this is slightly favorable to this baseline method when assessing pretraining performance (i.e., it eliminates the possibility of cross-corpus confusions, which is possible for the neural network methods). Correlation methods were performed only on the connectivity matrices from the full-duration scans (i.e., setting aside 100 s subsampled examples), with the exception of the analysis where correlation accuracy was assessed using the first 100 s sample of each validation scan.

The fully connected baseline method involved training models that comprised a single fully connected linear layer between the input connectivity matrices and the 4,987-unit output layer (i.e., no hidden or embedding layer). Otherwise, these models underwent the same training procedure as the shallow and deep models. Note that at a high level, this fully connected baseline approach is similar to connectome predictive modeling (Shen et al., 2017), except that we are using a fully connected linear neural network layer to learn the important edges for a given task. A neural network approach here also allows for a matched comparison with our shallow and deep models (and later with models that fine-tune brain2vec embeddings), because we can directly match hyperparameters (training data, learning rates, and epochs).

### Transfer-learning experiments: identification of new individuals

We used data from four held-out datasets (AOMIC PIOP1, AOMIC PIOP2, LA5c, and ds003592 which were not used during pretraining) to understand how generalizable our model representations were for distinguishing new participants and data. After preprocessing these fMRI data using the same pipeline above, we sent each (full-scan and subsampled) connectivity matrix through our pretrained models to extract the brain2vec model's embedding. Note that the models were frozen at this stage and the layers of the model were not updated during this step. For these four held-out datasets, we performed the same correlation-based fingerprinting experiments using the pretrained model embeddings to assess the fingerprinting capability of the model embeddings prior to any additional fine-tuning.

We then fine-tuned a linear readout layer from these embeddings to predict which individual a given training example belonged to, by learning a fully connected linear layer between the model embeddings and a new set of output units, one corresponding to each new participant in these datasets. Importantly, in this linear-readout experiment, we merely updated the mapping between the model embedding and a new set of output units corresponding to these new participants, while the rest of the model was held constant. Thus, successful identification of new subjects had to rely on information contained only within the model embeddings that were pretrained on the initial training corpus. These identification generalization experiments were carried out for each dataset individually. Participants in these datasets underwent multiple resting state or task scanning runs. We used cross-validation over scanning runs to assess model performance by iteratively holding out each of these runs for testing and training the fully connected output layer on the remaining runs. This fully connected layer was trained for 50 epochs using a cross-entropy training objective, stochastic gradient descent (SGD; momentum, 0.9; weight decay, 0.001) and a learning rate of 0.001. Again, we applied a class (i.e., subject) weighting in the objective to help offset any data imbalance (some participants had missing scans). After 50 epochs for learning the linear mapping between the embedding and the subject classes, the model was tested on how accurately it could recognize the individuals in this dataset based on the held-out scans. We used full-duration and subsampled scans as training examples but only tested on full-duration scans for the held-out runs. We evaluated above-chance performance (relative to the number of unique individuals in each dataset) using binomial tests, Bonferroni corrected for the number of runs in each dataset that we cross-validated over and the number of experiments we ran (152; 8 identification methods times 19 held-out scans).

Baseline methods were the same as those used for pretraining, only applied to these new datasets. The correlation method matched each held-out scan to the other scans in these datasets by calculating the correlation between the connectivity matrices and taking the subject ID corresponding to the scan with the highest correlation as the model's prediction. Again, this always involved the full-duration scans except when we used the 100 s duration scans as a test of this method under a limited duration constraint. This correlation method was also applied to the model embeddings for the held-out datasets to assess fingerprinting generalization prior to any fine-tuning. The fully connected baseline used the same training procedure as above, only here we replaced the model embedding as input to the fully connected layer with the corresponding connectivity matrices. Thus, this baseline learned a linear layer between the connectivity matrices and a set of output units corresponding to each participant. We trained the fully connected baseline on the same full-duration and subsampled training manifest that we used to fine-tune the brain2vec embedding.

### Transfer-learning experiments: predict traits and behavior

We used data from the same four held-out datasets (AOMIC PIOP1, AOMIC PIOP2, LA5c, and ds003592) not used during training to understand how generalizable our subject-recognition brain2vec representations were to new tasks. However, for these analyses cross-validation was carried out on the participant level rather than based on runs (i.e., fine-tuning using all the scanning runs from one set of participants and testing the model on all the scanning runs from an unseen set of participants). We took the same set of model embeddings (i.e., not updated since the initial pretraining phase) and conducted a new set of experiments where we trained new linear output layers to predict different variables reported in these data. Again, we conducted these within each dataset. For the PIOP datasets, we analyzed sex, handedness (categorical variables), Raven's cognitive test scores, age, and NEO personality variables (all continuous variables). Among the LA5c data, we analyzed gender, clinical diagnosis (both categorical variables), and age (continuous). Within ds003592, we analyzed each participant's young or old age grouping, study site, gender (all categorical), as well as age, cognitive (mostly NIH battery), big five personality variables (Big Five Aspects Scale), and MMSE (all continuous; Table 1), thus a similar set of cognitive dimensions as the PIOP datasets.

Fine-tuning to predict these trait and behavioral variables used iterative 10-fold cross-validation where the cross-validation folds were redrawn 10 separate times (thus 100-folds total). New, fully connected linear readout layers were trained on the scanning run data from 90% of participants. Testing was carried out on the scans from the held-out participants. Models were all trained for 10 epochs with a learning rate of 0.005, using SGD as above. We did not use subsampled scans for these experiments because there were many more training examples per output class. Experiments with discrete, categorical variables (e.g., gender, study site, diagnosis) were trained to minimize a cross-entropy loss function (again with class-wise weighting) with one output unit for each of the levels of a given variable (e.g., Site 1 or Site 2). Experiments with continuous variables (e.g., age, task performance, personality scales) were trained to minimize a mean squared error loss function with a single output unit. Since we tested on all scans for each participant, we assessed performance for each task for each scanning run. Regression performance was assessed by computing the correlation between predicted and observed values after pooling out-of-sample predictions across all 10 held-out participant folds within each cross-validation run. This yielded one aggregate correlation coefficient per cross-validation run (10 total), and these values were compared against zero using a one-sample Wilcoxon signed-rank test to assess whether performance exceeded chance. Classification performance was assessed in the same manner by pooling out-of-sample predictions across folds and computing an aggregate accuracy for each of the 10 cross-validation runs. The resulting accuracy values were compared against chance performance (50 or 25%, as appropriate) using one-sample Wilcoxon signed-rank tests to determine whether accuracy exceeded chance levels. We use false discovery rate correction for these analyses with the added criterion that the performance of all 10 cross-validation runs must exceed chance. The baseline, fully connected models were trained using the same procedure and again, the only difference was that the training procedure learned a linear layer between the original connectivity matrix and the output.

### Summary of data partitioning for fine-tuning experiments

Our fine-tuning experiments follow best practices from the speech technology field where benchmarks (e.g., SUPERB; Yang et al., 2021) often involve different data partitioning approaches. For diverse multitask benchmarking, data might need to be held out in different ways specific to a given downstream task such that a model's knowledge of intra- and interindividual variability can be appropriately challenged while mitigating data leakage between training (i.e., fine-tuning) and testing. Another standard approach to leakage mitigation that we follow is that all downstream tasks involve new datasets and participants not seen when pretraining the functional connectome fingerprinting models (specifically, as described above, LA5c, PIOP1, PIOP2, and ds003592 are held out from pretraining to support downstream fine-tuning and testing).

Our individual recognition fine-tuning experiments are set up similar to speaker identification tasks in the SUPERB benchmark (Nagrani et al., 2017; Yang et al., 2021) as well as other object recognition fine-tuning or transfer-learning tasks (Kornblith et al., 2019; Khosla et al., 2020; Kong et al., 2020). Here, a new closed set of targets (and thus output units) needs to be learned during fine-tuning using one set of examples and models are tested on new held-out examples of those targets. For individual recognition, different scanning sessions are held-out from fine-tuning to serve as test examples, and we cross-validate across scanning sessions such that each serves as a test case.

We perform an additional evaluation that implements the correlation method used by Finn et al. (2015), using both the raw connectivity matrices (as a baseline) and our pretrained model embeddings. This approach does not fine-tune a model but instead uses similarity or distance measures to assess whether a pair of examples comes from the same individual or not (analogous to the speaker verification task in SUPERB). For each new set of individuals in the datasets not seen during training, we take all the scans from a given task run and correlate the corresponding connectivity matrices or model embeddings with every scan in the remaining database of other task runs. If the scan in the database set with the highest correlation to the test scan is from the same individual, this is recorded as a match. Note that this method requires no additional updating or fine-tuning of the model or embeddings and is thus an unbiased view of how the model embeddings can help support individual identification in downstream data.

Finally, we evaluate our models by fine-tuning them for trait-based prediction tasks that exist across individuals (analogous to gender classification, speech transcription or emotion recognition, for example in SUPERB). Here, we fine-tune the model embeddings for each new task on all the data and scanning runs from a subset of participants and test on the data from a completely separate subset of *participants*. Leakage is mitigated for these tasks because testing is performed at the level of participants instead of runs (as in the earlier identification and verification tasks), and the model is evaluated on a subset of participants that is never seen during fine-tuning.

### Representational similarity analysis (RSA) to understand cognitive dimensions captured by model embeddings

RSA can offer another view into the information encoded in a representation, without explicitly defining a training objective. We ran each resting-state scan's full-duration connectivity matrix from ds003592 through our frozen, pretrained brain2vec models to obtain their embeddings. We then calculated the cosine distance between the embeddings derived from each subject's scans to obtain network-dissimilarity matrices. Similar functional connectome dissimilarity matrices were derived by calculating the cosine distance between the connectivity matrices that would be used as inputs to our models. Finally, we calculated the difference between each subject's score on different cognitive and personality measures within the dataset to obtain a set of behavior-dissimilarity matrices. Again, we focused on the cognitive and personality variables reported in ds003592 because there was substantial cognitive testing and metadata and the participant group spanned a wide age range. We calculated semipartial Spearman correlations between the behavior-dissimilarity matrices and the connectome and network-dissimilarity matrices to understand how the differences among individuals on these behavioral measures were captured by different representations of the models or neural data. These semipartial correlations allowed us to examine the relationship between, for example, a behavioral dissimilarity matrix and a model's dissimilarity matrix while statistically controlling for the rest of the behavioral variables. These RSA correlations were Bonferroni corrected as a function of the number of network (2) or connectome (2) dissimilarity matrices against the behavioral dissimilarity matrices (27; for 108 tests total). Ninety-five percent confidence intervals for each association were estimated using bootstrap resampling (1,000 times) over subjects. Due to dependencies inherent in pairwise dissimilarity matrices, these intervals may not align exactly with parametric *p* values derived from semipartial Spearman correlation tests. Only correlations that pass a Bonferroni’s correction and whose 95% bootstrapped confidence intervals exceed zero are discussed. We assessed only Session 1 scans but obtained a similar pattern of results based on the Session 2 scans. Similar analyses using standard (nonsemipartial) Spearman correlations are reported in Extended Data Figure 9-1. Finally, we restricted our analyses to participants who did not have any missing data among the behavioral measures we targeted, since this was a prerequisite of the semipartial correlation function we used.

## Results

### Pretraining neural networks for functional connectome fingerprinting

We compiled a pretraining dataset from six large fMRI datasets [Table 1; HCP1200, Van Essen et al. (2013); NKI-Rockland, Nooner et al. (2012); MPI-Leipzig Mind-Brain-Body, Babayan et al. (2019); Mendes et al. (2019); 1,000 Functional Connectomes, Biswal et al. (2010); AOMIC ID1000, Snoek et al. (2021); OASIS-3, LaMontagne et al. (2019)], which comprised data from 4,987 unique participants and 21,453 functional runs. These data were preprocessed to distill a functional connectivity matrix (netmat) based on the 100-node Schaefer atlas that was used as input to the model. Netmats for each full functional run along with netmats from 100 s subsampled segments of those runs were used as training examples (167,200 total training examples). During training, the model's objective was to correctly match each functional connectivity matrix with one of the corresponding 4,987 participant identifiers in the training dataset. We explored a variety of model parameters including standard or partial Pearson’s correlations for the input netmats, model depth (one or four hidden layers), model size (1,024, 512, 256 or 128 units in the penultimate hidden, or embedding, layer), and various dropout rates (0, 10, 25, and 50% dropout applied to the hidden layers; see Materials and Methods for complete pretraining details). To validate performance during training, we held out data from 1 d of scans in the cases where a participant came in for scans across multiple days (see Materials and Methods for further details on model pretraining). These validation scans were separated from the training scans by either days (HCP) or years (NKI, OASIS-3). We retained the model with the highest performance for both regular and partial correlation netmat inputs for further experiments. We refer to these as the “brain2vec” models.

**Figure 2.**
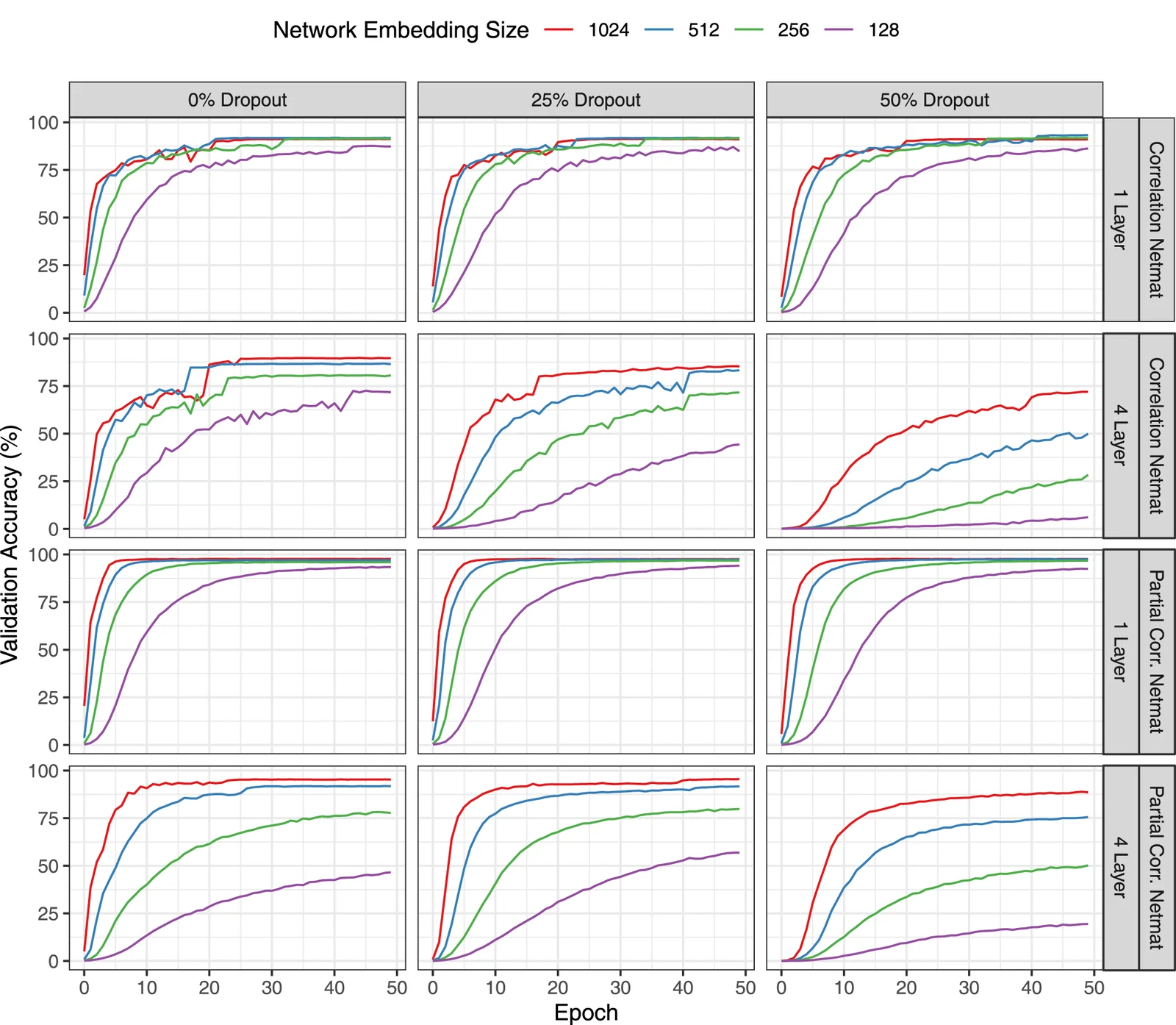
Brain2vec model performance for different hyperparameters assessed on the validation data across training epochs. See Extended Data Figures 2-1–2-8 for confusion matrices.

We were able to achieve high performance on the validation data (all greater than chance of 0.02% for a 4,987-way task, Bonferroni-corrected *p* < 0.001 unless otherwise specified) across a wide range of hyperparameters (Fig. 2; 25 models achieved >90% accuracy or at least as follows: accuracy = 91.10% CI = [90.61, 91.57]; precision = 0.872; recall = 0.858; F1 = 0.854; lowest-performing model: accuracy = 6.07% CI = [5.68, 6.48]; precision = 0.057; recall = 0.059; F1 = 0.048). In general, input netmats comprising partial correlations and larger embedding layer sizes improved performance, as did applying dropout to the hidden layers (though high dropout rates hurt performance for larger models). Somewhat surprisingly, a shallow architecture with one hidden layer outperformed the deeper architectures with four hidden layers. The best-performing model achieved 97.64% accuracy on the validation data overall (CI = [97.38, 97.89]; precision = 0.930; recall = 0.925; F1 = 0.921). This model comprised one 1,024-unit hidden layer with partial correlations as input and with no dropout applied to the hidden layers during training. Performance was highest for the HCP data (10,426 correct out of 10,442 scans from 1,061 participants; 99.85% accuracy CI = [99.75, 99.91]; precision = 0.999; recall = 0.998; F1 = 0.998) followed by NKI-Rockland (2,465 correct out of 2,608 scans from 535 participants; 94.52% accuracy CI = [93.57, 95.36]; precision = 0.913; recall = 0.897; F1 = 0.896), MPI-Leipzig (102 correct out of 109 scans from 109 participants; 93.58% accuracy CI = [87.22, 97.38]; precision = 0.883; recall = 0.887; F1 = 0.884), and OASIS-3 (433 correct out of 591 scans from 296 participants; 73.27% accuracy CI = [69.50, 76.79]; precision = 0.597; recall = 0.547; F1 = 0.558). The top-performing model using standard correlation netmats as input comprised one 512-unit hidden layer trained with 50% dropout and achieved performance that was lower than the best partial correlation model (on the validation data overall, 93.29% CI = [92.86, 93.71]; precision = 0.892; recall = 0.881; F1 = 0.877; on HCP, 95.02% CI = [94.59, 95.43]; precision = 0.948; recall = 0.944; F1 = 0.942; on NKI-Rockland, 90.34% CI = [89.14, 91.44]; precision = 0.818; recall = 0.782; F1 = 0.789; on MPI, 96.33% CI = [90.87, 98.99]; precision = 0.929; recall = 0.929; F1 = 0.929; and on OASIS-3, 75.30% CI = [71.61, 78.72]; precision = 0.661; recall = 0.630; F1 = 0.629). Confusion matrices for each best-performing model and each validation dataset can be found in Extended Data Figures 1-2–2-8. Where errors occurred the model predominantly confused participants from the same dataset, but a small number of scans were confused with participants from different datasets, particularly for OASIS-3.

10.1523/ENEURO.0370-25.2026.f2-1 Figure 2-1 Confusion matrix for the HCP validation dataset from the best-performing model taking partial correlation netmats as input. All errors are shown, however only a random subset of correct trials (along the diagonal) from these datasets is presented since the unfiltered data result in large n-by-n matrices with most of the points along the diagonal. Download Figure 2-1, TIF file.

10.1523/ENEURO.0370-25.2026.f2-2 Figure 2-2 Confusion matrix for the NKI validation dataset from the best-performing model taking partial correlation netmats as input. All errors are shown, however only a random subset of correct trials (along the diagonal) from these datasets is presented since the unfiltered data result in large n-by-n matrices with most of the points along the diagonal. Download Figure 2-2, TIF file.

**Figure 3.**
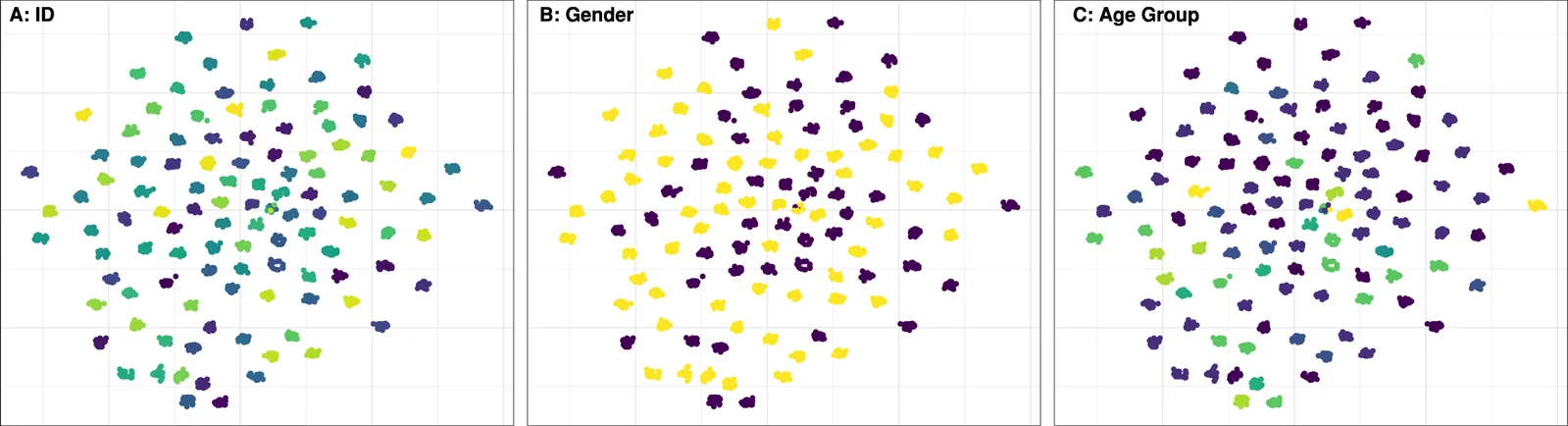
Partial correlation brain2vec model embeddings for the MPI validation data represented in a two-dimensional space via *t*-SNE (perplexity = 30, 1,000 max iterations). The same scatterplot of data from 109 participants is presented in panels ***A***, ***B***, and ***C***, but points are colored by subject ID (***A***), gender (***B***), and age-group in 5 year increments (***C***) to convey the information learned by the model. Points comprise both full-duration scans as well as the corresponding 100 s segments. Individuals with multiple clusters or stray points in this subspace indicate scans or time windows that may be more dissimilar from the rest of that participant's data and likely contribute to less-than-ceiling classification accuracy.

10.1523/ENEURO.0370-25.2026.f2-3 Figure 2-3 Confusion matrix for the entire MPI validation dataset from the best-performing model taking partial correlation netmats as input. Download Figure 2-3, TIF file.

**Figure 4.**
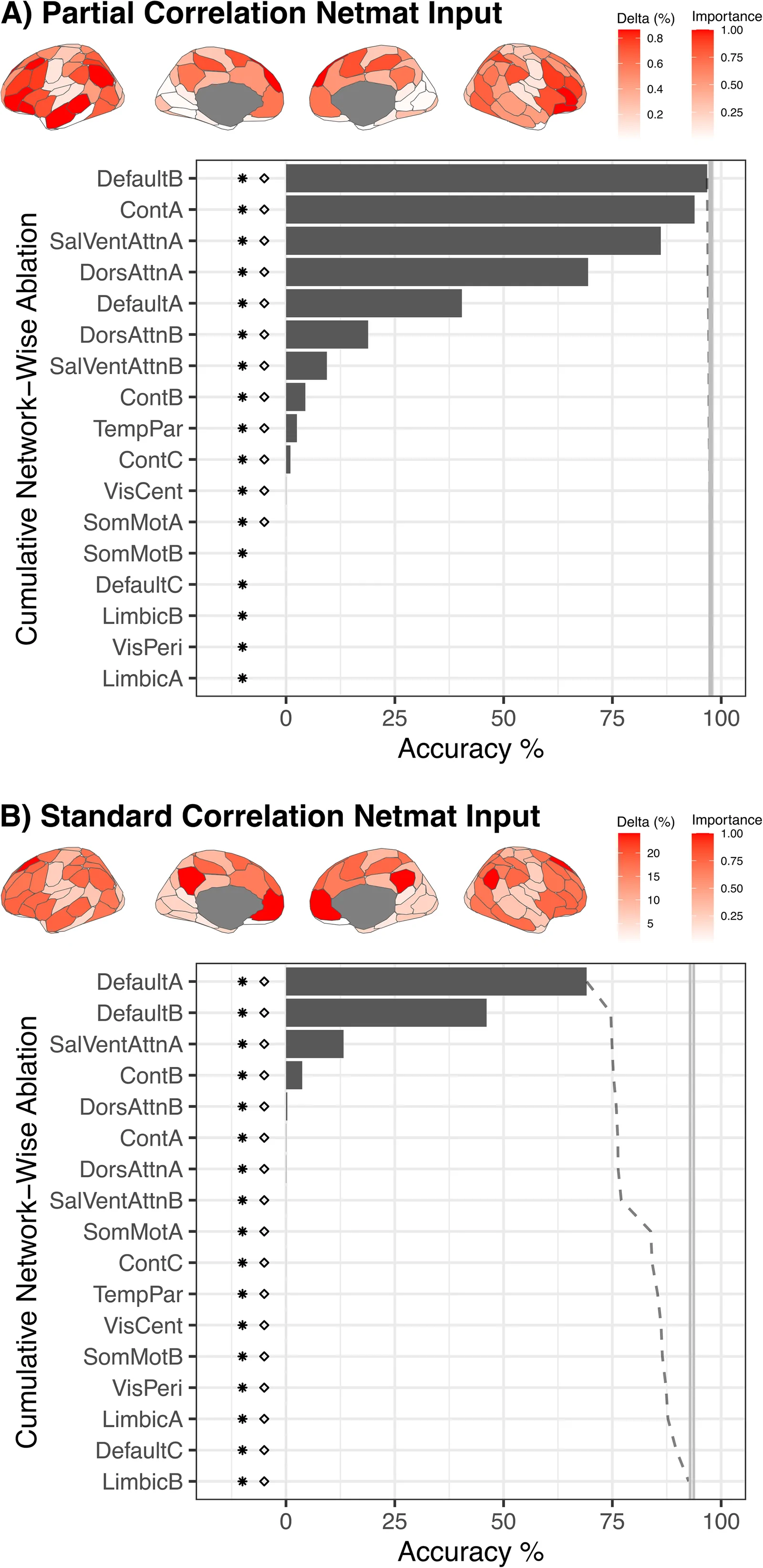
Ablation experiments indicating which networks had the largest impact on validation performance when they were removed for both partial (***A***) and standard correlation (***B***) netmat inputs. Reduction in validation performance is normalized to derive an overall importance score within each plot. Networks are ordered on the *y*-axis by how much they reduced recognition accuracy on their own in an initial pass (they are ordered by their ranked importance or delta scores from highest to lowest), with each network's individual ablation accuracy indicated by dotted lines. Networks that resulted in a statistically significant reduction in accuracy when removed individually are indicated by diamonds (*p* < 0.05 binomial exact test on the discordant paired outcomes of each ablated model relative to the original model). Gray solid lines indicate 95% confidence intervals around accuracy performance for the original, unablated model (***A***, 97.61% CI = [97.35, 97.86]; ***B***, 93.29% CI = [92.86, 93.71]). Asterisks indicate networks whose cumulative ablated input connectivity matrix significantly reduced accuracy relative to the unablated model (*p* < 0.05 binomial exact test on the discordant paired outcomes of each ablated model relative to the original model). Similar analyses based on individual network nodes can be found in the supplemental material (Extended Data Figs. 4-1, 4-2).

10.1523/ENEURO.0370-25.2026.f2-4 Figure 2-4 Confusion matrix for the entire OASIS-3 validation dataset from the best-performing model taking partial correlation netmats as input. Download Figure 2-4, TIF file.

**Figure 5.**
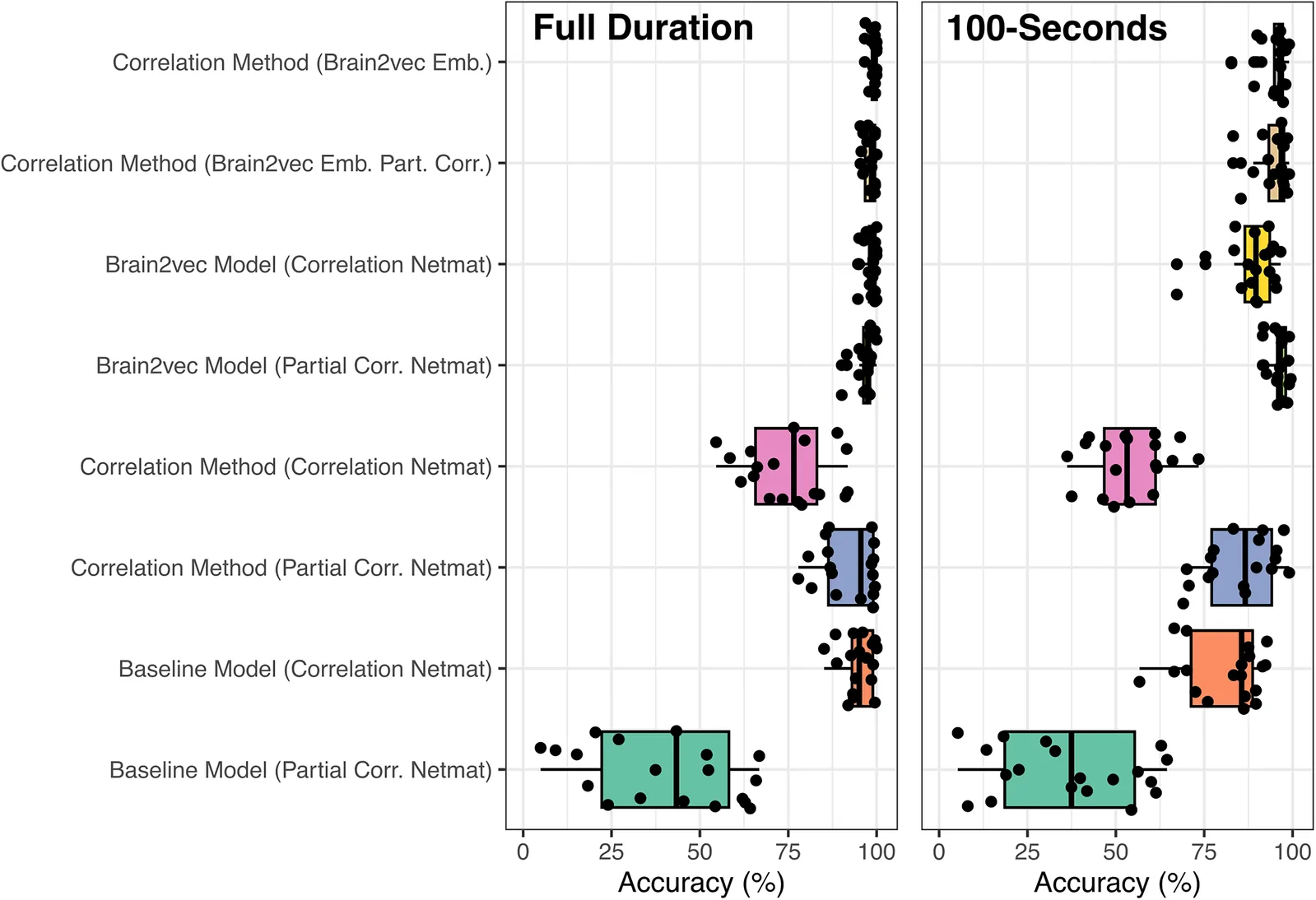
Performance of each identification method on recognizing individuals from new datasets aggregated across all held-out scans and datasets. Left, Results based on evaluations of held-out scans at their full duration. Right, Results based on evaluations of held-out scans when limited to just a 100 s duration. See Extended Data Figures 5-1–5-16 for confusion matrices.

10.1523/ENEURO.0370-25.2026.f2-5 Figure 2-5 Confusion matrix for the HCP validation dataset from the best-performing model taking standard correlation netmats as input. All errors are shown, however only a random subset of correct trials (along the diagonal) from these datasets is presented since the unfiltered data result in large n-by-n matrices with most of the points along the diagonal. Download Figure 2-5, TIF file.

**Figure 6.**
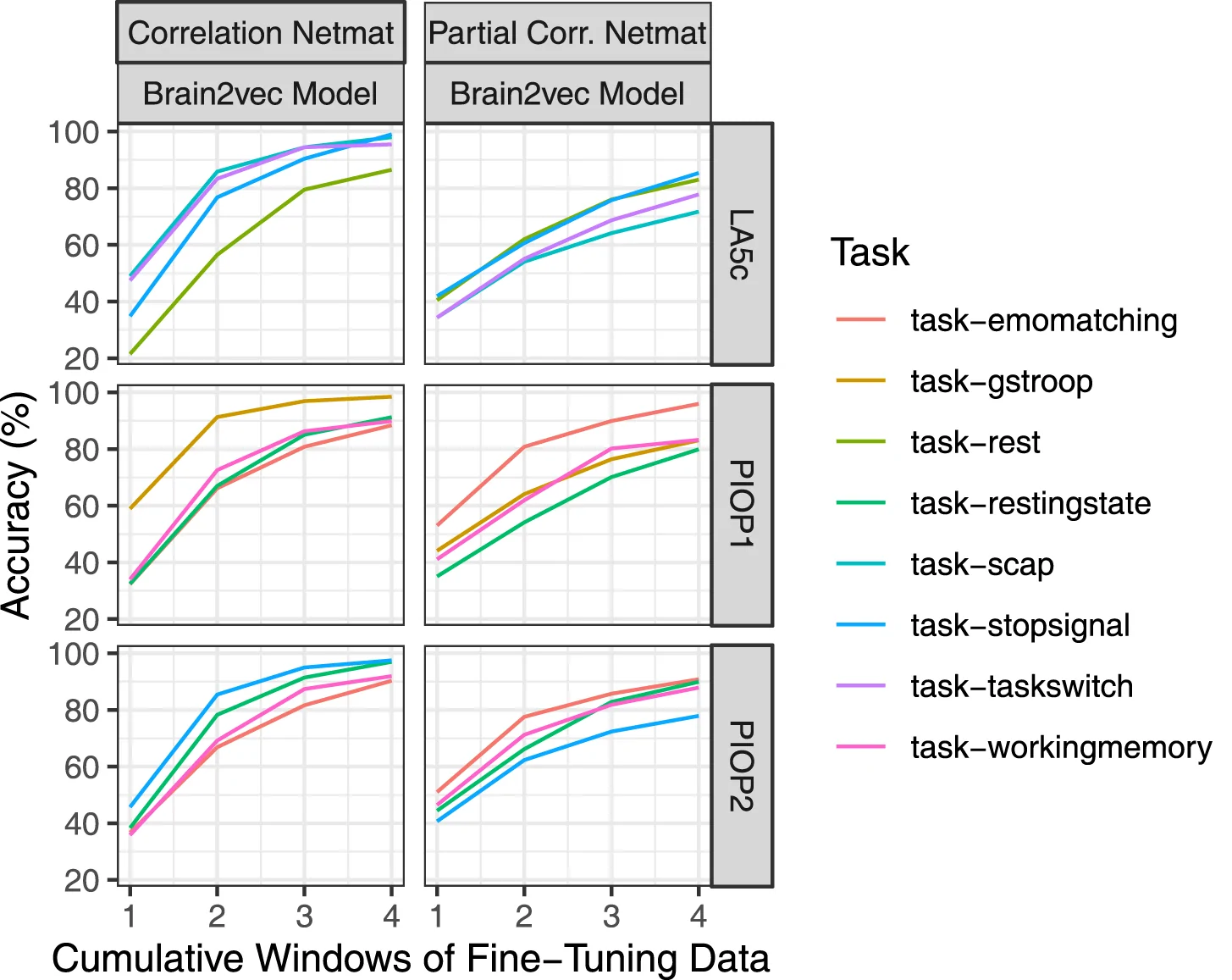
Performance of each brain2vec model recognizing individuals from new datasets as a function of the volume of data available for fine-tuning. Datasets in these experiments were yoked to comprise four tasks each and only 200 participants. Larger quantities of data for fine-tuning were strongly associated with improved recognition performance (*r_s_* = 0.84; *p* < 0.001).

10.1523/ENEURO.0370-25.2026.f2-6 Figure 2-6 Confusion matrix for the NKI validation dataset from the best-performing model taking standard correlation netmats as input. All errors are shown, however only a random subset of correct trials (along the diagonal) from these datasets is presented since the unfiltered data result in large n-by-n matrices with most of the points along the diagonal. Download Figure 2-6, TIF file.

**Figure 7.**
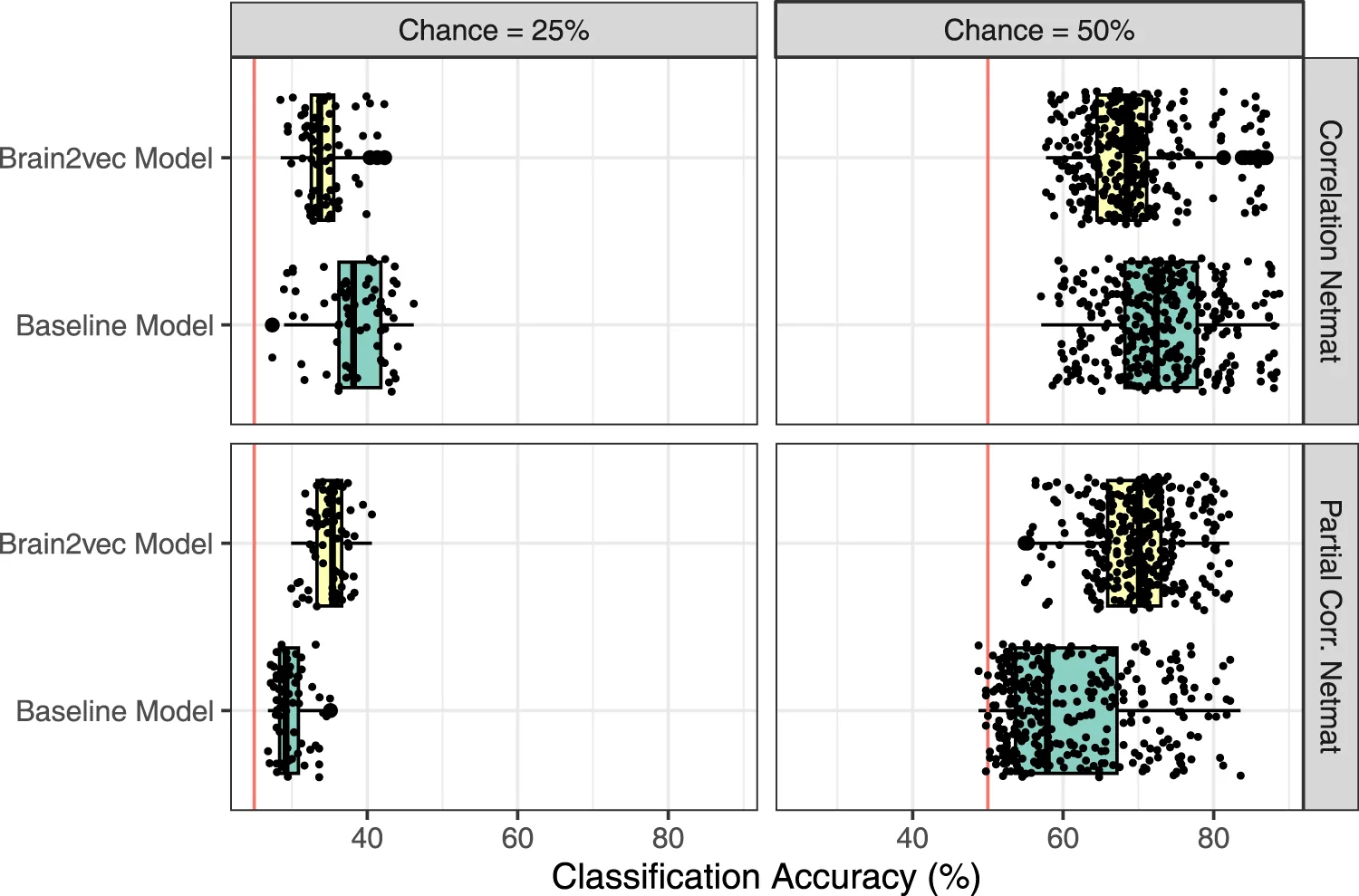
Performance of each identification method predicting categorical variables in new datasets aggregated across all held-out scans and datasets, displayed by chance level. Red lines indicate chance performance. Each dot represents an entire 10-fold cross-validation for each predicted variable or task (with 10 cross-validation runs, or dots, per variable). Extended Data Figure 7-1 provides task-level performance distributions across cross-validation runs for each model along with task-level statistical assessments.

10.1523/ENEURO.0370-25.2026.f2-7 Figure 2-7 Confusion matrix for the entire MPI validation dataset from the best-performing model taking standard correlation netmats as input. Download Figure 2-7, TIF file.

**Figure 8.**
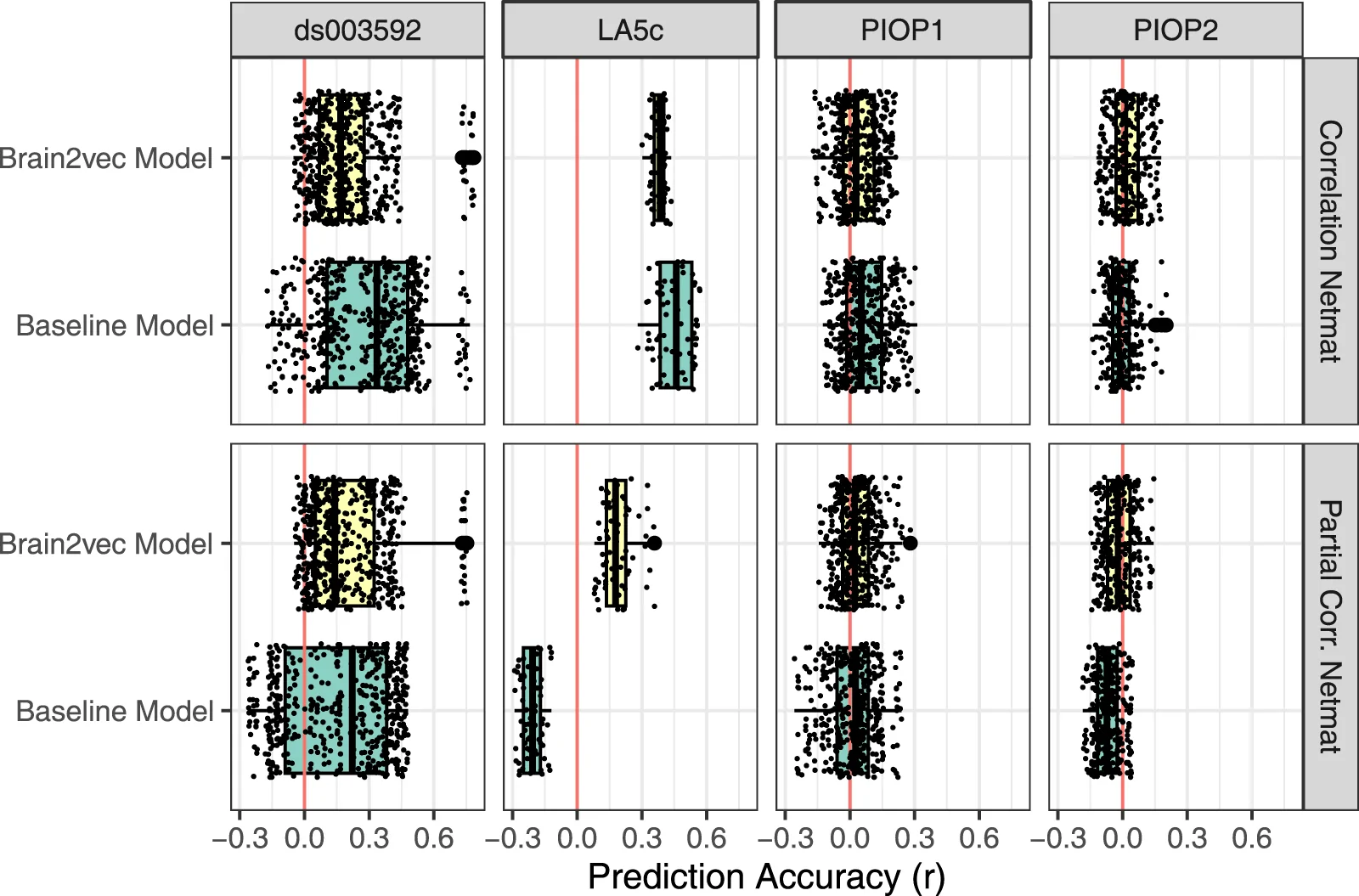
Performance of each identification method predicting continuous variables in new datasets aggregated across all held-out scans, displayed by dataset. Red lines indicate chance performance (*r* = 0 no correlation between model predictions and true values). Each dot represents an entire 10-fold cross-validation for each predicted variable or task (with 10 cross-validation runs, or dots, per variable). Extended Data Figures 8-1–8-4 provide task-level performance distributions across cross-validation runs for each model along with task-level statistical assessments.

10.1523/ENEURO.0370-25.2026.f2-8 Figure 2-8 Confusion matrix for the entire OASIS-3 validation dataset from the best-performing model taking standard correlation netmats as input. Download Figure 2-8, TIF file.

The rich embedding space of the top-performing partial correlation model is presented in Figure 3 using the MPI validation partition. Distinct clusters can be seen for individuals, but alignment with other trait-like features such as gender and age is limited. We quantified the separability of these features using silhouette scores, a measure of cluster quality comparing within- and between-cluster distances. This analysis found high scores for participants (silhouette score = 0.78), but lower scores for gender (silhouette score = 0.01) and age-group (silhouette score = −0.34).

Performance of the best model (using partial correlation netmat inputs) was very high on the HCP validation data even though the held-out HCP scans comprised many new tasks not seen during training. This form of generalization is a challenging task for a subject-recognition model since it requires learning features that pertain to individuals despite fMRI data being modulated by the performance of different, unseen tasks. High performance on these scans suggests the model has learned a high-level, generalizable representation of individuals. This high performance on novel tasks could also be due to the high volume of HCP data used in training (many scans, each fairly long), where the scans used for training also involved diverse tasks. Model performance for the best partial correlation input model was nearly the same, only 0.03% less accurate overall, when evaluation was performed on the first 100 s of the validation scans rather than their original 5–15 min duration (on the validation data overall, 97.61% CI = [97.35, 97.86]; precision = 0.930; recall = 0.925; F1 = 0.921; on HCP, 99.82% CI = [99.72, 99.89]; precision = 0.997; recall = 0.997; F1 = 0.997; on NKI-Rockland, 93.86% CI = [92.87, 94.75]; precision = 0.899; recall = 0.879; F1 = 0.880; on MPI, 94.50% CI = [88.40, 97.95]; precision = 0.911; recall = 0.920; F1 = 0.914; and on OASIS-3, 75.80% CI = [72.14, 79.20]; precision = 0.660; recall = 0.633; F1 = 0.630). The top-performing standard correlation input model fared worse, almost 5% less accurate overall, when data were limited to 100 s (on the validation data overall, 88.87% CI = [88.33, 89.39]; precision = 0.825; recall = 0.809; F1 = 0.805; on HCP, 90.74% CI = [90.17, 91.29]; precision = 0.902; recall = 0.891; F1 = 0.889; on NKI-Rockland, 86.73% CI = [85.37, 88.01]; precision = 0.744; recall = 0.698; F1 = 0.708; on MPI, 93.58% CI = [87.22, 97.38]; precision = 0.895; recall = 0.895; F1 = 0.895 and on OASIS-3, 64.47% CI = [60.46, 68.33]; precision = 0.515; recall = 0.484; F1 = 0.483).

#### Ablation analysis: localizing identifiability networks

We wanted to understand which brain regions our models relied on to distinguish individuals. To do this, we carried out an ablation analysis where we re-evaluated the validation data while we iterated through each of the 17 networks in the Schaefer brain atlas (Yeo et al., 2011; Schaefer et al., 2018), censoring (i.e., zeroing out) each of the networks in the input connectivity matrices in turn. We recorded how much validation accuracy decreased as each network was removed and converted this to a normalized importance score (similar to Lee et al., 2022; see Materials and Methods for details). Ablation analysis results are presented in Figure 4. This revealed that removing frontoparietal networks (specifically Yeo networks DefaultB, ContA, SalVentAttnA, and DorsAttnA) involving the default mode and attention networks incurred the largest reduction in participant recognition accuracy (96.78% CI = [96.47, 97.07]; Δ = −0.87%; 96.86% CI = [96.55, 97.14]; Δ = −0.79%; 96.92% CI = [96.61, 97.20]; Δ = −0.73 and 96.97% CI = [96.67, 97.25]; Δ = −0.68%, respectively, all binomial exact tests on discordant paired outcomes suggesting the original model performed better *p* < 0.05) for the best-performing model that relied on partial correlation netmats as input. However, we note that overall, the partial correlation model was robust to censoring individual networks. Accuracies changed only slightly (changes in accuracy of at most <1%) for each network that was removed (see Byrge and Kennedy, 2019 for related discussion). This suggests that this model learned a globally distributed representation of identifiability. Nonetheless, the importance of frontoparietal networks supports previous fingerprinting results that were able to obtain high accuracy when focusing on these areas (Finn et al., 2015). Overall, a similar pattern was observed implicating the importance of default mode and attention networks for the best standard correlation netmat input model, albeit with larger deltas when each network was censored (most influential Yeo networks: DefaultA, DefaultB, SalVentAttnA, and ContB, decreased validation performance 69.09% CI = [68.31, 69.86]; Δ = −24.20%; 74.63% CI = [73.90, 75.36]; Δ = −18.66%; 74.91% CI = [74.18, 75.63]; Δ = −18.39 and 75.25% CI = [74.52, 75.97]; Δ = −18.04%, respectively, all binomial exact tests on discordant paired outcomes suggesting the original model performed better; *p* < 0.05), suggesting a less global, more localized representation of identifiability for that model.

10.1523/ENEURO.0370-25.2026.f4-1 Figure 4-1 Ablation experiments indicating which nodes had the largest impact on validation performance when they were removed for partial correlation netmat inputs. Networks are ordered on the y-axis by how much they reduced recognition accuracy on their own in an initial pass (they are ordered by their ranked importance or delta scores from highest to lowest), with each node’s individual ablation accuracy indicated by dotted lines. Nodes that resulted in a statistically significant reduction in accuracy when removed individually are indicated by diamonds (*p* < 0.05 binomial exact test on the discordant paired outcomes of each ablated model relative to the original model). Grey solid lines indicate 95% confidence-intervals around accuracy performance for the original, unablated model (97.61% CI [97.35, 97.86]). Asterisks indicate networks whose cumulative ablated input connectivity matrix significantly reduced accuracy relative to the un-ablated model (*p* < 0.05 binomial exact test on the discordant paired outcomes of each ablated model relative to the original model). Download Figure 4-1, TIF file.

10.1523/ENEURO.0370-25.2026.f4-2 Figure 4-2 Ablation experiments indicating which nodes had the largest impact on validation performance when they were removed for standard correlation netmat inputs. Networks are ordered on the y-axis by how much they reduced recognition accuracy on their own in an initial pass (they are ordered by their ranked importance or delta scores from highest to lowest), with each node’s individual ablation accuracy indicated by dotted lines. Nodes that resulted in a statistically significant reduction in accuracy when removed individually are indicated by diamonds (*p* < 0.05 binomial exact test on the discordant paired outcomes of each ablated model relative to the original model). Grey solid lines indicate 95% confidence-intervals around accuracy performance for the original, unablated model (93.29% CI [92.86, 93.71]). Asterisks indicate networks whose cumulative ablated input connectivity matrix significantly reduced accuracy relative to the un-ablated model (*p* < 0.05 binomial exact test on the discordant paired outcomes of each ablated model relative to the original model). Download Figure 4-2, TIF file.

To further probe the importance of different brain networks for identification performance and to better understand how robust the partial correlation model was to the removal of input networks, we ran a second analysis where networks were cumulatively ablated from the input. That is, instead of ablating networks individually, we now ablated the networks in series, in order of their individual reduction in performance (highest to lowest delta/importance scores), zeroing out more of the input until the entire input connectivity matrix was removed. While the removal of an individual network still had a small but significant impact on the validation performance of the partial correlation input model (96.78% CI = [96.47, 97.07]; Δ = −0.87%; binomial exact test *p* < 0.05), by the time four networks were removed, performance dropped below 75% (69.42% CI = [68.64, 70.19]; Δ = −28.23%; binomial exact test *p* < 0.05), and performance dropped below 50% (40.4% CI = [39.58, 41.23]; Δ = −57.24%; binomial exact test *p* < 0.05) after five networks were removed. The model that used standard correlation input netmats was again less robust with a more localized representation that dropped below 75% after one network was removed (69.09% CI = [68.31, 69.86]; Δ = −24.20%; binomial exact test *p* < 0.05) and below 50% (46.09% CI = [45.26, 46.93]; Δ = −47.20%; binomial exact test *p* < 0.05) after a second network was removed. Similar analyses based on individual network nodes can be found in the supplemental material (Extended Data Figs. 4-1, 4-2), although the removal of individual nodes produced in many cases very small changes in performance.

#### Control experiments

Neural networks are efficient and powerful learners, and it is difficult to completely rule out whether a model has learned to exploit artifacts in the training data to improve performance. We conducted multiple follow-up control experiments to examine the influence of common fMRI artifacts in these data and found they could not explain our model's performance.

If the models are exploiting motion artifacts unique to individuals, a model trained directly on these artifacts should perform well at distinguishing individuals. However, replacing our fMRI netmat training samples with their corresponding motion confounds (mean and standard deviation of *x*, *y*, *z* and their derivatives over the corresponding time windows) achieved <4% accuracy on held-out days of scans (3.72% CI = [3.41, 4.05]; precision = 0.021; recall = 0.020; F1 = 0.017; *p* < 0.001 binomial test compared with chance performance; and *p* < 0.001 binomial exact test on the discordant paired outcomes of the motion confound model relative to the original model), suggesting that motion is not driving these results.

Similarly, if the models are learning to exploit artifacts from anatomical normalization, then spatially filtering the data (a common preprocessing step to mitigate the influence of anatomical artifacts) should reduce performance. Filtering the functional data with an 8 mm spatial filter did not result in an appreciable change in validation accuracy for partial correlation netmat input models (validation partition accuracy, 97.89% CI = [97.64, 98.12]; precision = 0.937; recall = 0.930; F1 = 0.928; *p* < 0.001 binomial test compared with chance performance and *p* = 0.004 binomial exact test on the discordant paired outcomes of the spatially filtered model relative to the original model) but did incur a slight reduction in the performance of standard correlation netmat input models (validation partition accuracy, 89.80% CI = [89.28, 90.30]; precision = 0.852; recall = 0.842; F1 = 0.835; *p* < 0.001 binomial test compared with chance performance and *p* < 0.001 binomial exact test on the discordant paired outcomes of the spatially filtered model relative to the original model). Overall, these control experiments suggest that artifacts from anatomical normalization are not driving these results.

#### Comparison with baseline methods

Finally, our brain2vec model's performance compares favorably to other methods. For example, correlation-based fingerprinting methods do not perform well on large datasets using partial correlation netmats from the same 100-node parcellation (on the validation data overall, 54.96% CI = [54.12, 55.79]; precision = 0.707; recall = 0.552; F1 = 0.559; *p* < 0.001 binomial test compared with chance performance and *p* < 0.001 binomial test on discordant paired outcomes relative to the original model; on HCP, 50.56% CI = [49.59, 51.52]; precision = 0.737; recall = 0.499; F1 = 0.510; on NKI-Rockland, 68.52% CI = [66.70, 70.30]; precision = 0.670; recall = 0.560; F1 = 0.572; on MPI, 89.91% CI = [82.66, 94.85]; precision = 0.844; recall = 0.860; F1 = 0.849; and on OASIS-3, 66.50% CI = [62.53, 70.30]; precision = 0.636; recall = 0.603; F1 = 0.593) but can perform better on smaller datasets (MPI) or those comprised of mostly resting-state scans (NKI-Rockland and OASIS-3). The accuracy of the correlation method decreases considerably when using standard correlation matrices comprised of 100 nodes (overall validation accuracy, 30.51% CI = [29.74, 31.29]; precision = 0.406; recall = 0.305; F1 = 0.318; *p* < 0.001 binomial compared with chance performance and *p* < 0.001 binomial test on discordant paired outcomes relative to the original model).

As an additional baseline, we trained a linear readout layer (i.e., a fully connected linear neural network layer between the input and an output layer with no hidden nodes) mapping from the input correlation matrices to the 4,987 output units (one for each subject identifier) using our full training dataset. This is essentially a neural implementation of connectome predictive modeling, where a fully connected neural network layer is used to learn the most relevant edges in the input connectivity matrices for predicting participant IDs based on the training data. This method also performed well on our validation data, albeit performing below our best-performing partial correlation netmat input model (validation partition accuracy, 96.90% CI = [96.60, 97.19]; precision = 0.936; recall = 0.918; F1 = 0.920; and using standard correlation netmat inputs validation partition accuracy, 74.60% CI = [73.87, 75.33]; precision = 0.723; recall = 0.685; F1 = 0.682; all *p* < 0.001 binomial test against chance performance and *p* < 0.001 binomial exact test on the discordant paired outcomes of the motion confounds model relative to the original model) and worse on the 100 s validation scans (95.63% CI = [95.27, 95.96]; precision = 0.900; recall = 0.873; F1 = 0.878 and 62.58% CI = [61.76, 63.39]; precision = 0.597; recall = 0.544; F1 = 0.544 using partial and standard correlation inputs, respectively). These baseline analyses indicate that the functional connectivity matrices we used as input to our model are very rich features for distinguishing individuals and mapping individual variability. However, neither of these baseline methods produces a generalizable representation that can bring insights that might be learned from the population during pretraining to bear on new tasks.

### Generalizing model representations to identify new participants from new datasets

An extensible resource in neuroimaging will need to achieve high performance on new datasets containing individuals and tasks not seen during training. Moreover, a useful transfer-learning resource should bring along insights learned from large-scale pretraining to aid in analyses of new data.

As a first test of how well our functional connectome fingerprinting model's representations generalize, we conducted the correlation-based fingerprinting analysis developed by Finn et al. (2015) for four new datasets. This is analogous to a speaker verification transfer-learning benchmark task (Yang et al., 2021), where unseen speakers are compared using pretrained model representation distances. This analysis compared fingerprinting performance based on both the 100-node network connectivity matrices and the embeddings from the model we pretrained. Prior to any fine-tuning, this analysis will tell us how well the embeddings we generated in pretraining can be adapted to new, limited datasets. We used data from four new datasets, each containing at least 200 participants and multiple functional scanning runs: AOMIC PIOP1 and PIOP2 (Snoek et al., 2021), LA5c (Poldrack et al., 2016), and Setton et al. (2023; Spreng et al., 2022; openneuro.org number ds003592). As shown in Figure 5 (top two rows), the pretrained model embeddings were able to match participants across different scanning runs with near-ceiling accuracy (for partial correlation input model LA5c, 96.51% CI = [95.53, 97.32]; precision = 0.963; recall = 0.959; F1 = 0.957; on PIOP1, 98.95% CI = [98.21, 99.44]; precision = 0.964; recall = 0.966; F1 = 0.964; on PIOP2, 99.66% CI = [99.02, 99.93]; precision = 0.997; recall = 0.997; F1 = 0.996; on ds003592, 98.59% CI = [97.25, 99.39]; precision = 0.981; recall = 0.984; F1 = 0.982; for the standard correlation input model LA5c, 99.24% CI = [98.71, 99.60]; precision = 0.986; recall = 0.985; F1 = 0.985; on PIOP1, 99.19% CI = [98.52, 99.61]; precision = 0.966; recall = 0.968; F1 = 0.966; on PIOP2, 99.89% CI = [99.38, 99.99]; precision = 0.999; recall = 0.999; F1 = 0.999; on ds003592, 97.36% CI = [95.69, 98.52]; precision = 0.964; recall = 0.972; F1 = 0.965; all *p* < 0.001 Bonferroni-corrected binomial tests against chance performance; see supplemental material, Extended Data Figs. 5-1–5-8 for confusion matrices). Importantly, this evaluation required no fine-tuning or adaptation on downstream participants, demonstrating that the pretrained embeddings themselves encode identity-related structure that generalizes to entirely unseen datasets and individuals. Both models outperformed baseline correlation-based fingerprinting analyses run using their respective connectivity matrices (Fig. 5 rows five and six; paired Wilcoxon signed-rank test of fingerprinting performance using partial correlation embeddings vs connectivity matrices: *V* = 4; *p* < 0.001; and using standard correlation embeddings vs connectivity matrices, *V* = 0; *p* < 0.001). This suggests good generalizability for these models to new data, even prior to any fine-tuning or adaptation.

10.1523/ENEURO.0370-25.2026.f5-1 Figure 5-1 Confusion matrix for the entire LA5c dataset from the best-performing model taking partial correlation netmats as input using the correlation method. Download Figure 5-1, TIF file.

10.1523/ENEURO.0370-25.2026.f5-2 Figure 5-2 Confusion matrix for the entire PIOP1 dataset from the best-performing model taking partial correlation netmats as input using the correlation method. Download Figure 5-2, TIF file.

10.1523/ENEURO.0370-25.2026.f5-3 Figure 5-3 Confusion matrix for the entire PIOP2 dataset from the best-performing model taking partial correlation netmats as input using the correlation method. Download Figure 5-3, TIF file.

10.1523/ENEURO.0370-25.2026.f5-4 Figure 5-4 Confusion matrix for the entire ds003592 dataset from the best-performing model taking partial correlation netmats as input using the correlation method. Download Figure 5-4, TIF file.

10.1523/ENEURO.0370-25.2026.f5-5 Figure 5-5 Confusion matrix for the entire LA5c dataset from the best-performing model taking standard correlation netmats as input using the correlation method. Download Figure 5-5, TIF file.

10.1523/ENEURO.0370-25.2026.f5-6 Figure 5-6 Confusion matrix for the entire PIOP1 dataset from the best-performing model taking standard correlation netmats as input using the correlation method. Download Figure 5-6, TIF file.

10.1523/ENEURO.0370-25.2026.f5-7 Figure 5-7 Confusion matrix for the entire PIOP2 dataset from the best-performing model taking standard correlation netmats as input using the correlation method. Download Figure 5-7, TIF file.

10.1523/ENEURO.0370-25.2026.f5-8 Figure 5-8 Confusion matrix for the entire ds003592 dataset from the best-performing model taking standard correlation netmats as input using the correlation method. Download Figure 5-8, TIF file.

**Figure 9.**
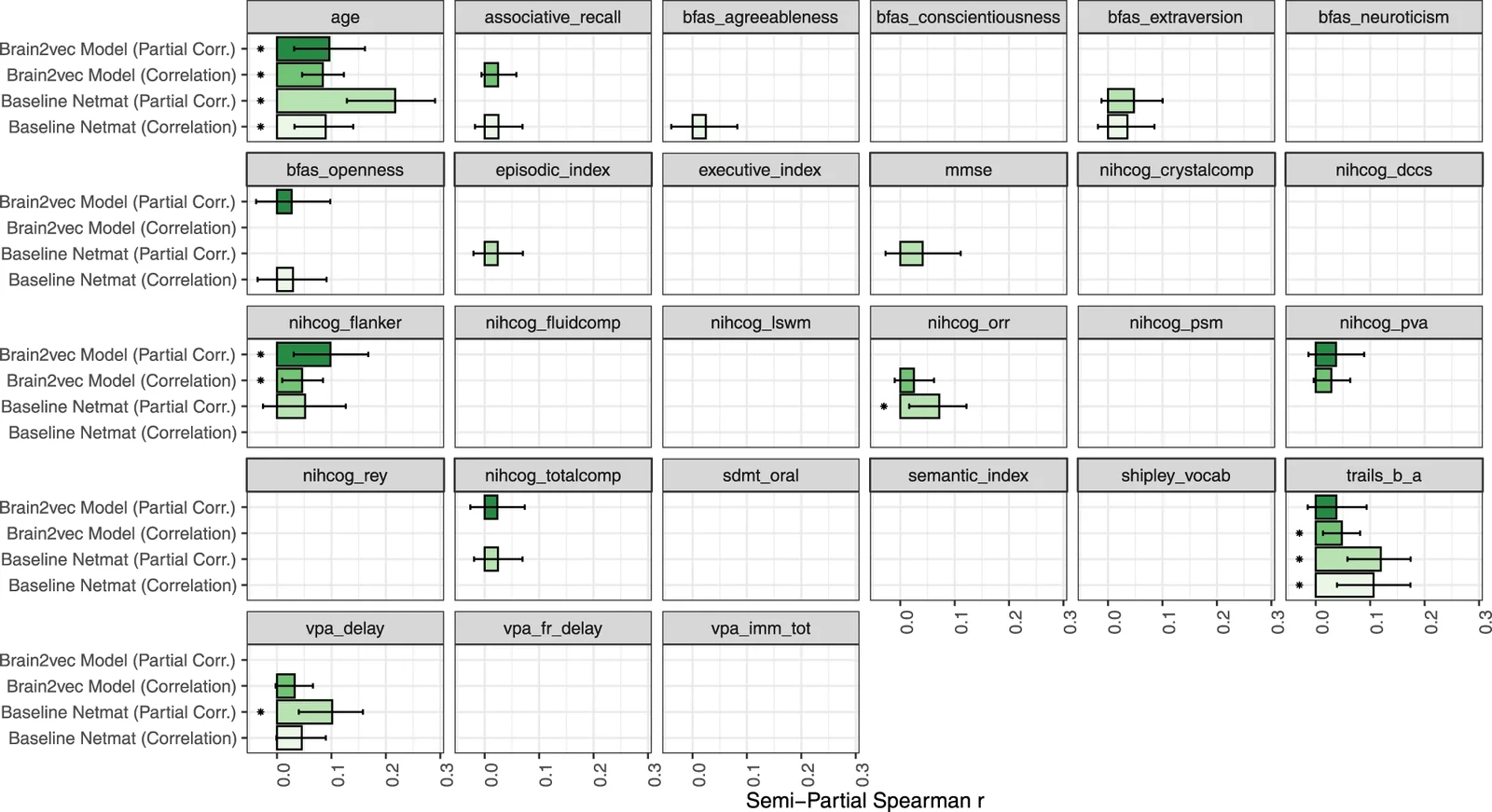
Significant semipartial correlations between behavioral and neural representations (dissimilarity matrices) after holding all other features constant. Each bar represents a semipartial correlation statistic that can be interpreted as the association between each behavioral and neural variable after statistically controlling for all the others (e.g., the association between the Flanker task and the partial correlation fingerprinting model after controlling for age, associative recall, and all other behavioral measures). Only tests that survived a Bonferroni’s correction are displayed, and only those whose 95% bootstrap confidence intervals exceed zero are starred and discussed. Extended Data Figure 9-1 provides the standard (nonsemipartial) Spearman correlations between behavioral and neural representations.

10.1523/ENEURO.0370-25.2026.f5-9 Figure 5-9 Confusion matrix for the entire LA5c dataset from the best-performing model taking partial correlation netmats as input using the fine-tuning method. Download Figure 5-9, TIF file.

10.1523/ENEURO.0370-25.2026.f5-10 Figure 5-10 Confusion matrix for the entire PIOP1 dataset from the best-performing model taking partial correlation netmats as input using the fine-tuning method. Download Figure 5-10, TIF file.

10.1523/ENEURO.0370-25.2026.f5-11 Figure 5-11 Confusion matrix for the entire PIOP2 dataset from the best-performing model taking partial correlation netmats as input using the fine-tuning method. Download Figure 5-11, TIF file.

10.1523/ENEURO.0370-25.2026.f5-12 Figure 5-12 Confusion matrix for the entire ds003592 dataset from the best-performing model taking partial correlation netmats as input using the fine-tuning method. Download Figure 5-12, TIF file.

10.1523/ENEURO.0370-25.2026.f5-13 Figure 5-13 Confusion matrix for the entire LA5c dataset from the best-performing model taking standard correlation netmats as input using the fine-tuning method. Download Figure 5-13, TIF file.

10.1523/ENEURO.0370-25.2026.f5-14 Figure 5-14 Confusion matrix for the entire PIOP1 dataset from the best-performing model taking standard correlation netmats as input using the fine-tuning method. Download Figure 5-14, TIF file.

10.1523/ENEURO.0370-25.2026.f5-15 Figure 5-15 Confusion matrix for the entire PIOP2 dataset from the best-performing model taking standard correlation netmats as input using the fine-tuning method. Download Figure 5-15, TIF file.

10.1523/ENEURO.0370-25.2026.f5-16 Figure 5-16 Confusion matrix for the entire ds003592 dataset from the best-performing model taking standard correlation netmats as input using the fine-tuning method. Download Figure 5-16, TIF file.

Next, we conducted a series of transfer-learning experiments. For each dataset, we trained a new fully connected output layer to classify these new individuals based on the pretrained fingerprinting models' frozen embeddings (i.e., one new output unit per participant in the new dataset, replacing the old set of 4,987 output units). This is analogous to a speaker identification transfer-learning benchmark (Yang et al., 2021), where, during fine-tuning, a new output layer is learned to identify a new closed set of speakers using one set of examples and the model is then tested on recognizing those speakers using a separate unseen set of examples. Each dataset contained multiple scanning runs, often involving different tasks, which we used for cross-validation. A new fully connected output layer was trained (for 50 epochs) using data from all runs except one which we held out for testing, iterating through each task run as the held-out run in turn. We recorded how accurately the model could identify the participants of a given dataset based on each held-out run.

Our model's representations supported very accurate individual identification on these new datasets (above-chance identification on all held-out scans, Bonferroni-corrected *p* < 0.001; Fig. 5). Representations from the best-performing partial correlation input model supported an average of 96.86% identification accuracy for these new participants across all held-out scans and datasets (LA5c, 97.61% CI = [96.77, 98.28]; precision = 0.980; recall = 0.976; F1 = 0.976; on PIOP1: 98.46% CI = [97.60, 99.07]; precision = 0.987; recall = 0.981; F1 = 0.982; on PIOP2: 96.20% CI = [94.73, 97.36]; precision = 0.969; recall = 0.960; F1 = 0.960; on ds003592: 90.85% CI = [88.17, 93.09]; precision = 0.923; recall = 0.908; F1 = 0.900; all *p* < 0.001 Bonferroni-corrected binomial tests against chance performance), while representations from the best-performing standard correlation input model supported slightly better (paired-sample Wilcoxon signed-rank test: *V* = 17; *p* = 0.003) performance with an average of 98.53% accuracy on these new data (LA5c, 99.48% CI = [99.01, 99.76]; precision = 0.995; recall = 0.994; F1 = 0.994; on PIOP1, 98.70% CI = [97.90, 99.25]; precision = 0.984; recall = 0.982; F1 = 0.982; on PIOP2, 98.44% CI = [97.39, 99.14]; precision = 0.987; recall = 0.985; F1 = 0.984; on ds003592, 94.89% CI = [92.75, 96.55]; precision = 0.951; recall = 0.949; F1 = 0.942; all *p* < 0.001 Bonferroni-corrected binomial tests against chance performance). Datasets with more data available for fine-tuning (i.e., a higher number of scans for fine-tuning) achieved better identification performance, with accuracy on PIOP1 ranging from 97 to 100% across held-out scans and accuracy on LA5c ranging from 96 to 100%. Performance was slightly lower on ds003592 (90.14–95.07%). Low performance on ds003592 could have resulted from limited scanning data or a less diverse set of task runs (all resting state) available for fine-tuning. In any case, the high performance we observed on the PIOP and LA5c datasets is notable, in that this requires model representations to generalize well across a wide set of tasks, many of which were not seen during training. This high performance matches or exceeds performance on our original validation data (see previous section) indicating the representation our model learned is generalizable to new participants and scanning protocols. Confusion matrices for each best-performing model on each held-out dataset can be found in the supplemental material (Extended Data Figs. 5-9–5-16).

To better understand how fine-tuning data volumes contributed to these results, we ran an additional experiment where we constrained the volume of fine-tuning data for three downstream datasets with multiple tasks (LA5c, PIOP1, and PIOP2). We constrained each dataset to have a comparable number of tasks (four tasks each, “rest,” “scap,” “stopsignal,” “taskswitch” for LA5c; “emomatching,” “restingstate,” “workingmemory,” “gstroop” for PIOP1 and “emomatching,” “restingstate,” “workingmemory,” “stopsignal” for PIOP2) and limited them to a maximum of 200 participants for this experiment. Then, using cross-validation over tasks, we fine-tuned a new output layer to recognize these individuals using only one 100 s window of data per task, and we progressively increased the amount of training data in subsequent fine-tuning runs up to four 100 s windows per task. The results of these data scaling experiments can be seen in Figure 6. Performance strongly correlated with additional data across models, tasks, and datasets (*r_s_* = 0.84; *p* < 0.001), meaning that a larger volume of pretraining data improves downstream performance. In some cases, just four 100 s segments of data were sufficient to reach near-ceiling performance, particularly for models trained on standard correlation netmat inputs.

To understand how beneficial our pretraining strategy was and whether the intermediate representation of our models encoded useful information for these fine-tuning tasks, we trained a linear readout layer to identify participants in each dataset directly from the input netmat correlation matrices using the same set up above (i.e., again holding out each task scanning run in turn). Note that because our model embeddings are much smaller (512 or 1,024 dimensions) than these connectivity matrices (4,950 dimensions), this allows the fully connected baseline models to have many times more free parameters to perform these tasks. All the fully connected baseline models performed better than chance recognizing individuals from held-out scans (Fig. 5 lines seven and eight; Bonferroni-corrected *p* < 0.001). Both of our brain2vec models outperformed their respective fully connected baselines (paired-sample Wilcoxon signed-rank test for partial correlation input model mean = 96.86% vs fully connected baseline mean = 39.94%: *V* = 190; *p* < 0.001; and for standard correlation input model mean = 98.53% vs fully connected baseline mean = 95.00%: *V* = 168; *p* < 0.001). The partial correlation input fully connected baseline model performed quite poorly with accuracy ranging from 7.04% overall on ds003592 to 61.15% overall on LA5c, while its counterpart based on standard correlation input netmats performed quite well (88.55% on ds003592 to 97.83% on LA5c).

Finally, this pattern of results was consistent, and the brain2vec models further outperformed their respective baselines, when test scans in these held-out datasets were limited to a 100 s duration (Fig. 5). While all 100 s duration experiments still outperform chance (Bonferroni-corrected *p* < 0.001), performance of all methods based on standard correlation decreased substantially compared with full-duration evaluations. Overall average performance of the brain2vec model based on standard correlation input netmats decreased from 98.53 to 88.63%. Meanwhile the overall average performance of the brain2vec model based on partial correlation input netmats is robust to this truncated scan duration (96.86–96.40%). Nonetheless, both brain2vec models outperformed all their respective baselines when duration of the evaluation scans is limited (paired-sample Wilcoxon signed-rank tests, all *p* < 0.001; Fig. 5), indicating that pretraining is effective in encoding information from a wider base of participants that improves performance on downstream tasks over an already-powerful netmat representation.

### Generalizing model embeddings to predict traits

Individual variability in functional connectivity patterns have been linked to differences in behavior, traits, and cognition (Rosenberg et al., 2016, 2020; Greene et al., 2018). However, interindividual variability likely comprises a large number of cognitive and neural dimensions, and it would be difficult to achieve an adequate sample of the population that can model the relevant brain–behavior dynamics within a single study (Marek et al., 2022). Thus, a powerful application for a generalizable functional fingerprinting resource would be to map the latent space of interindividual variability within a large sample of the population that encodes relevant features such that the representation could be deployed to model brain–behavior associations for new data and applications. If our model has adequately mapped and encoded this latent space, the learned representations may contain information useful for predicting behavioral performance and individual traits in new datasets. To test this, we ran a new set of transfer-learning experiments to predict participant traits or performance on several different cognitive tasks, using the same held-out datasets (AOMIC PIOP1 and PIOP2, LA5c, and ds003592). Again, we froze the network and trained a new fully connected layer to predict traits and behavioral measures based on our model's embeddings. In these experiments, the output layers were trained to predict subject metadata other than their IDs (see Materials and Methods for details). Different from the identification experiments that used session- or scan-wise data partitions, these analyses used participant-wise data partitioning schemes where the model was fine-tuned on all the scans from a subset of participants and then the model was tested on the scans from a separate set of participants. This is analogous to tasks in the SUPERB speech benchmark that recognize targets that exist across specific individuals (such as speech transcription or emotion recognition). These analyses used a 10-fold cross-validation scheme where the model was fine-tuned on data from 90% of participants and was tested on 10% of participants such that each 10% fold served as a test case. The 10-fold partitions were redrawn over 10 separate cross-validation runs (thus 100 folds total), and results were averaged over the testing partitions for each run which formed a distribution we compared against chance performance. These experiments involved learning both regression (e.g., for predicting age or performance on a cognitive task) and classification tasks (e.g., for predicting gender or disease status), thus depending on the nature of the data and task the output units and training objectives were updated accordingly (to a single output unit trained to optimize mean squared error or an output unit for each class trained with cross entropy, respectively). Because different tasks performed in the scanner could modulate brain activity in a way that might emphasize useful features of individual variability (Finn et al., 2017; Gratton et al., 2018) or task performance (Greene et al., 2018), we fine-tuned the model using all available scan data (here, full-duration scans only) for a given subject in the training fold. When evaluating the held-out participant's data, we assess model performance for each measure as a function of the in-scanner task the participant performed.

In general, our brain2vec model performance on these nonidentification tasks was lower than for recognizing large numbers of new individuals (for which our model's performance was near ceiling), but our models could still predict many task outcome variables well above chance. The best-performing pretrained model that took partial correlation netmat inputs significantly predicted all of the 40 classification variables (Fig. 7) above chance (false discovery rate-corrected *p* < 0.05, Wilcoxon signed-rank test that the observed distribution exceeded chance; see Extended Data Fig. 7-1 for a breakdown by task, dataset, and model), but performance was modest (median = 68.88%). Performance was lower for regression tasks, and the model significantly predicted 79 of 131 regression variables better than chance (Fig. 8; all false discovery rate-corrected *p* < 0.05; see Extended Data Figs. 8-1–8-4 for a breakdown by task, dataset, and model). Its counterpart that took correlation netmat inputs performed similarly for classification tasks (false discovery rate-corrected *p* < 0.05, Wilcoxon signed rank test that the observed distribution exceeded chance), albeit with lower accuracy overall (median = 66.97%) but significantly predicted 90 of 131 regression variables above chance (all false discovery rate-corrected *p* < 0.05).

10.1523/ENEURO.0370-25.2026.f7-1 Figure 7-1 Task, model, run and data-level breakdown of fine-tuning results for classification tasks. Each row conveys data-set aggregated accuracy for each cross-validation run as individual points, summarized by a boxplot. Asterisks indicate experiments that exceeded chance performance comparing all cross-validation runs to chance using FDR-corrected Wilcoxon signed-rank test, and requiring all cross-validation runs to exceed chance. Download Figure 7-1, TIF file.

10.1523/ENEURO.0370-25.2026.f8-1 Figure 8-1 Task, model, run and data-level breakdown of fine-tuning results for regression tasks in the LA5c dataset. Each row conveys data-set aggregated accuracy (Pearson correlation between model outputs and reference values) for each cross-validation run as individual points, summarized by a boxplot. Asterisks indicate experiments that exceeded chance performance comparing all cross-validation runs to chance using FDR-corrected Wilcoxon signed-rank test, and requiring all cross-validation runs to exceed chance. Download Figure 8-1, TIF file.

10.1523/ENEURO.0370-25.2026.f8-2 Figure 8-2 Task, model, run and data-level breakdown of fine-tuning results for regression tasks in the PIOP1 dataset. Each row conveys data-set aggregated accuracy (Pearson correlation between model outputs and reference values) for each cross-validation run as individual points, summarized by a boxplot. Asterisks indicate experiments that exceeded chance performance comparing all cross-validation runs to chance using FDR-corrected Wilcoxon signed-rank test, and requiring all cross-validation runs to exceed chance. Download Figure 8-2, TIF file.

10.1523/ENEURO.0370-25.2026.f8-3 Figure 8-3 Task, model, run and data-level breakdown of fine-tuning results for regression tasks in the PIOP2 dataset. Each row conveys data-set aggregated accuracy (Pearson correlation between model outputs and reference values) for each cross-validation run as individual points, summarized by a boxplot. Asterisks indicate experiments that exceeded chance performance comparing all cross-validation runs to chance using FDR-corrected Wilcoxon signed-rank test, and requiring all cross-validation runs to exceed chance. Download Figure 8-3, TIF file.

10.1523/ENEURO.0370-25.2026.f8-4 Figure 8-4 Task, model, run and data-level breakdown of fine-tuning results for regression tasks in the ds003592 dataset. Each row conveys data-set aggregated accuracy (Pearson correlation between model outputs and reference values) for each cross-validation run as individual points, summarized by a boxplot. Asterisks indicate experiments that exceeded chance performance comparing all cross-validation runs to chance using FDR-corrected Wilcoxon signed-rank test, and requiring all cross-validation runs to exceed chance. Download Figure 8-4, TIF file.

Our models clearly performed best on classification tasks. Neither model was able to very accurately predict continuous variables in the PIOP1 and PIOP2 datasets, where performance was typically low (less than *r* = 0.20) and often (for almost half the conditions tested) did not significantly exceed chance (though classification performance on those datasets was similar to other datasets). Models performed best on the ds003592 data, accurately predicting study site (86.30%, CI = [85.64, 86.97]) and age-group (80.70%, CI = [79.93, 81.34]) as well as age (e.g., *r* = 0.78; CI = [0.77, 0.78]; and 11.84 MAE CI = [11.60, 12.11] for the model using standard correlation netmat inputs). We were also able to significantly predict participant performance on different memory and cognitive variables (albeit to a lower degree of accuracy) including performance on the Verbal Paired Association test (delay; *r* = 0.41; CI = [0.39, 0.43]), Associative Recall Paradigm (*r* = 0.45; CI = [0.43, 0.46]), Symbol Digits Modality Task (*r* = 0.32; CI = [0.29, 0.34]), and Trails Making Task (*r* = 0.41; CI = [0.40, 0.43]; all false discovery rate-corrected *p* < 0.05).

We wanted to determine if our embeddings and pretraining procedure distilled insights from the larger population they were trained on about traits and cognitive performance beyond what could be gleaned from the (already quite powerful) connectivity matrices we used as input. To do this, we trained a series of baseline, fully connected models for comparison. These models underwent the same procedure, training a single fully connected layer to predict traits and cognitive variables (evaluated on held-out participants using the same cross-validation procedure) from the flattened connectivity matrices instead of using our model's frozen, pretrained embedding as input. Again, note that our model embeddings have a much smaller dimensionality (512 or 1,024 dimensions) than the full connectivity matrices (4,950 dimensions) and thus have many fewer free parameters for these fine-tuning experiments. Overall, there was only a small but statistically significant increase in performance from our models vs the baseline models for the categorical, classification tasks (paired-sample Wilcoxon signed-rank test: *V* = 111,145; *p* < 0.001) and for the continuous, regression tasks (paired-sample Wilcoxon signed-rank test: *V* = 1,819,990; *p* = 0.008). This was because the results for each input feature space were mixed. Our pretrained model that took partial correlation netmats as input outperformed its fully connected netmat baseline model (paired-sample Wilcoxon signed-rank test for classification tasks: *V* = 4,836; *p* < 0.001; and for regression tasks: *V* = 321,278; *p* < 0.001), but the pretrained model that took Pearson’s correlation netmat inputs performed significantly worse than its baseline (paired-sample Wilcoxon signed-rank test for classification tasks: *V* = 62,426; *p* < 0.001; and for regression tasks: *V* = 600,101; *p* < 0.001)

### RSA to understand cognitive dimensions captured by model embeddings

Probe tasks and fine-tuning are a useful way to understand the information encoded in a model representation (Raj et al., 2019; Peri et al., 2020) and demonstrate potential utility for new applications, but these involve many choices including hyperparameters, training targets, and optimizations. RSA can provide another view into the information encoded by a network embedding that is less biased by optimization goals and tuning parameters (Kriegeskorte and Kievit, 2013; Mehrer et al., 2020; Ogg and Skerritt-Davis, 2021; Ogg et al., 2026). We used the extensive cognitive and behavioral measures obtained by Setton et al. (2023), Spreng et al. (2022), and colleagues in ds003592 to carry out a RSA that would examine what participant traits or cognitive features our network encoded. We used semipartial correlations for this analysis so we could assess the unique correlation for each trait or cognitive feature.

Correlations among the network and functional connectome dissimilarity matrices showed only a weak to moderate association with one another. The partial and standard correlation brain2vec model embedding representations were moderately correlated (*r_s_* = 0.315; *p* < 0.001) with one another, and these models were moderately correlated with their respective input feature spaces (*r_s_* = 0.289 and *r_s_* = 0.484, between netmats and model embeddings that took partial and standard correlation netmats, respectively, both *p* < 0.001). The partial and standard correlation netmat representations were also moderately correlated (*r_s_* = 0.346; *p* < 0.001). This suggests that the pretraining process produces a latent space that differs from the original input space and that different netmat features encode information differently from one another, which could be useful in differential utility for downstream tasks.

Finally, we wanted to understand how the latent space of our models and the input network connectivity matrices encoded traits and cognitive features of individuals. We computed semipartial correlations between each neural and behavioral dissimilarity matrix to isolate the association with each behavioral measure while statistically controlling for all the others. These results are presented in Figure 9 (Extended Data Fig. 9-1 conveys the nonsemipartial correlations between each neural and behavioral representation). The strongest associations across the neural and behavioral variables were for age (semipartial *r_s_* = 0.096; bootstrap CI = [0.031, 0.162]; Bonferroni-corrected *p* < 0.05) and the flanker task (semipartial *r_s_* = 0.098; bootstrap CI = [0.031, 0.168]; Bonferroni-corrected *p* < 0.05) as well as the VPA (semipartial *r_s_* = 0.101; bootstrap CI = [0.040, 0.158]; Bonferroni-corrected *p* < 0.05) and trail-making task (semipartial *r_s_* = 0.120; bootstrap CI = [0.058, 0.174]; Bonferroni-corrected *p* < 0.05) for the connectivity matrices. Most of these associations were common across both the model and netmat features, but the amount by which these representational spaces correlated with behavioral features differed. Notably, many associations between behavioral and neural representations persisted even after statistically controlling for age, which likely introduced some bias related to the cognitive and memory measures in our fine-tuning experiments.

10.1523/ENEURO.0370-25.2026.f9-1 Figure 9-1 Significant non-semi-partial Spearman correlations between behavioral and neural representations (dissimilarity matrices). Each bar represents a correlation statistic that can be interpreted as the association between each behavioral and neural variable. Only tests that survived a Bonferroni correction are displayed and only those whose 95% bootstrap confidence intervals exceed zero are starred and discussed. Download Figure 9-1, TIF file.

## Discussion

Neuroimaging data have increased in scale, inviting important questions regarding whether these data can be harnessed to distill generalizable representations that could be used for downstream clinical or psychological applications or to gain new research insights. However, this may require a very large number of participants (Button et al., 2013; Helwegen et al., 2023). Here, we sought to pretrain a model of neural data that could provide a representation of brain function that is aware of the high-dimensional space of individual variability, so that downstream or more data limited applications could perform better with respect to these challenges. Similar insights have been gleaned in other domains like text (Devlin et al., 2018), speech (Amodei et al., 2016), and vision (Krizhevsky et al., 2012) by combining large datasets and pretraining neural network models. This approach has also been successful in other areas of neuroscience (Leonardsen et al., 2022; Ogg and Coon, 2024; Ogg et al., 2025; Coon and Ogg, 2026), but generalizable deep-learning applications for fMRI have been less well studied, likely due to issues stemming from the diversity of scanning protocols or the high cost of acquiring MRI (Szucs and Ioannidis, 2020).

Our work constitutes a key step toward generalizable representations for fMRI. Where many fMRI applications may be challenged by the large individual variability that exists among population samples (Gratton et al., 2018; Seghier and Price, 2018), we sought to leverage this variability as a resource for generalization. Similarly, we hope our large-scale, pretrained representation could distill information that could be useful for smaller datasets where it may be difficult or impossible (e.g., in the case of rare diseases) to get the large sample sizes that may be needed for brain-wide association studies (Marek et al., 2022). The results presented here make some progress on these fronts.

We trained neural networks to recognize a high number of individuals from functional connectivity matrices, achieving high performance among held-out scans from known participants. More importantly, the model's representation could be used to distinguish entirely new participants from datasets not used during pretraining. Finally, this model supported above-chance prediction on new tasks beyond distinguishing individuals, albeit at reduced accuracy. However, these initial results are encouraging and may invite further exploration in pretraining and fine-tuning models across datasets in fMRI.

Functional connectome fingerprinting has been an active area of research (Finn et al., 2015; Amico and Goñi, 2018; Abbas et al., 2020; Griffa et al., 2022), but these studies have primarily involved modest numbers of individuals from single high-quality datasets. Neural network applications in this area are also not new (Wang et al., 2019; Misra et al., 2021; Sarar et al., 2021), but again, these studies have not looked at repurposing the intermediate representation of these models for new data. We achieve near-ceiling accuracy on multiple datasets, even when scan duration is severely limited. However, we note that accuracy among older populations (e.g., the OASIS-3 data) leaves room for improvement. Since we were investigating a large-scale problem, we used all available data, regardless of scanner task, but it will be interesting to see if certain tasks or procedures can emphasize individual variability during pretraining or for improving fine-tuning [see Finn et al. (2017) for discussion] or if automated quality control approaches (which is itself an active area of research; Taylor et al., 2023) to triage data before pretraining further improves performance or generalization. We broaden the scope of prior work by integrating multiple datasets and creating transferable representations that can encode individual variability to support recognition on new datasets. One interesting observation was that the correlation method we used as a baseline does not scale well to large datasets with thousands of individuals and diverse scanning runs. However, matching scans from individuals across sessions by taking the highest correlation is effective when data are limited to a few hundred participants, and this method is especially effective when the task is a resting-state scan in both the fine-tuning and test dataset, as has also been pointed out using a subset of the HCP data (Finn et al., 2015). Here, we demonstrate this on a large scale across more datasets (compare correlation method performance for ds003592, 98.9–99.3%, to performance on LA5c, 77.9–87.4%). We observed the reverse pattern when training a linear readout layer to classify participants directly from correlation matrices with no hidden layers: this method performed very well when data are plentiful (e.g., among the large set of participants in pretraining, 96.9% validation accuracy), but this approach can fail when data are limited (e.g., when training was limited to each of the held-out datasets, 7–61.3% on the identification transfer-learning experiments). Our method of training a generalizable representation performs well both when data are abundant and when it is limited and also supports accurate individual identification when generalizing across datasets. Finally, our functional connectome fingerprinting models rely on frontoparietal networks (e.g., the default mode network) and implicated a similar set of regions for individual variability as in previous work (Finn et al., 2015).

Our primary goal in this work was to try and develop a transferable representation for different tasks. Our approach aligns with broader trends in the field, including the prediction of new states (Finn and Constable, 2016; Woo et al., 2017; Poldrack et al., 2020; Finn and Rosenberg, 2021). We partially succeed on this front. The representation learned by our model was most useful for supporting tasks similar to those used for pretraining (distinguishing individuals within new datasets) but was not as successful in other nonsubject-recognition tasks. For example, our models could be fine-tuned to significantly predict one of four classes of psychiatric disease within the LA5c dataset (31–39% accuracy), but this is far from ceiling and was not as accurate in some cases as a baseline model (as high as 43% accurate). We note that this reduced performance may at least partially stem from a domain shift. Since the model was pretrained on a large quantity of healthy individuals, connectivity patterns associated with different neuropsychiatric conditions were likely out-of-domain for our model. Nonetheless, our model could also be used to significantly predict some cognitive and memory performance variables (e.g., VPA, Associative Recall, Trail Making within ds003592, but not within the PIOP data), reasonably accurately (*r* = 0.27–0.45), within the range of some past efforts (Greene et al., 2018) but performing lower than others (Finn et al., 2015; Rosenberg et al., 2016). Nonetheless, our results suggest that for some tasks, even transformations learned via these shallow neural networks can learn a compact representation of a high-dimensional latent space that is generalizable to new tasks and data. In fact, our shallow network that performed well in this work is similar to early NLP architectures (e.g., word2vec; Mikolov et al., 2013). In our fine-tuning experiments, this means our model representations (comprising 512 or 1,024 dimensions) often performed as well as or better than the baseline models (with 4,950 dimensions) using far fewer free parameters.

In the course of this work, we made the decision to pretrain the model on diverse datasets for neural fingerprinting. This borrowed from other fields [e.g., speech recognition, whisper, Radford et al. (2023); sleep stage classification, *U*-sleep, Perslev et al. (2021); and image recognition, DINOv2; Oquab et al., 2023], where robust off-the-shelf performance for later users is enabled by diverse pretraining data. We used as input to our model an already-powerful network connectivity matrix representation that summarizes a window of a time series allowing for more harmonization across disparate scanning protocols. This intermediate representation made it possible to homogenize (at least to some degree) the data from different studies so they could be compiled for a large pretraining run. Indeed, we see very little confusion between pretraining datasets and can later accurately classify study site in ds003592, suggesting the model learned features related to sites and perhaps different scanners. While a narrow focus on neural fingerprinting could invite criticism that this approach involves shortcut learning, we believe this is an acceptable tradeoff for two reasons. First, the usefulness or impact of such a shortcut is likely limited because there are only a handful of sites and datasets in pretraining and still thousands of participants per site in some cases. Learning information about sites in this case does not trivialize performance in ultimately distinguishing the hundreds or thousands of participants, it must still distinguish per site. Second, awareness of scanner and dataset diversity helps ensure the model is robust and useful in downstream tasks or for new data. Because there is some representation of these features embedded within what the model has learned, it does not fail when it encounters new datasets. Rather, it has some bearing with how to embed them within its latent space. The very high downstream performance (which is our main focus) observed for neural fingerprinting suggests this is the case.

Age is encoded in our model's representations and has a large influence on some of these downstream tasks, but it does not explain all the variance captured by our model. For example, AOMIC PIOP1 and PIOP2 have limited age ranges, and our model could support accurate recognition of these individuals and other categorical demographic features. Our model also performed well among the HCP participants, who are all young adults and fall within a limited age range. Our RSA also highlights that our model encodes features related to executive functioning and memory, even when partialing out variance related to age. Still, age was well predicted by fine-tuning our model, and accuracy on downstream tasks that were not related to subject identification was lower, emphasizing the need for more work. Future work should better understand how features are encoded in these neural network layers and how to reweight features learned in an initial pretraining round. Indeed, since shallow architectures performed best in pretraining most of our experiments focused on these, an interesting approach would be to only freeze or fine-tune some layers of deeper models.

This work and the goal of pretrained representations that can be helpful for downstream tasks has implications for privacy considerations. On the one hand, a model that learns representations of traits is useful for new data where samples may be limited (such as for rare diseases). On the other hand, as a research exercise in understanding individual variability, there may be interest in a pure representation of the individual that is agnostic to these traits. Our results suggest that large-scale fingerprint pretraining strongly supports generalization for identity-related tasks, while transfer to broader behavioral and cognitive prediction tasks is more modest and variable. Ultimately if one is successful on the main goal of trait prediction, it may be desirable to remove nontrait features of individual variability to preserve privacy. Similar concerns may also apply to self-supervised approaches in MRI (Caro et al., 2023; d’Ascoli et al., 2025; Yang et al., 2025) and beyond (Chen et al., 2022; Ogg and Coon, 2024; Coon and Ogg, 2026) where individual variability can be a dominant feature of the structure that models learn from the data. Adversarial post-training could be useful to remove variability along with nuanced benchmarking to quantify the balance between useful trait and identification learning (e.g., a benchmark of predictive tasks where the goal is for performance to be high but with included identifiability tasks where it might be desirable for performance to be low).

One interesting finding from our work was that shallow networks tended to perform the identification task better than deeper networks. It is likely that the input connectivity matrices are already very powerful, so little additional learning may be required on top of these, though the RSA analyses indicate these representations are indeed different from one another. Nonetheless, other architectures might better handle these data and distill richer or more interpretable representations. This could involve graph neural networks or sequence learning models that can learn temporal features across subsampled connectivity netmats in consecutive windows. We are eager for future work in these areas that build on the findings presented here.

There are numerous other avenues well suited to follow-up work, and we are eager to see where this field heads. First, traveling-subject data, where the same cohort of individuals is imaged by multiple scanners would be very illuminating and could help adjudicate issues of data or scanner bias, how sensitive (or not) model representations are to these features, and how they might interact with downstream prediction tasks. Also, optimizing functional scanning runs for emphasizing individual variability or to align with downstream prediction targets is an area ripe for further study. There is a predominance of resting-state data available, but it has been suggested that these paradigms could be too unconstrained (Finn and Rosenberg, 2021), whereas an activity like movie watching might allow for a more homogenous baseline of neural activity that could act as a carrier for features related to individual variability (Misra et al., 2021). This work will also generally benefit from new datasets for testing model representations, and we are particularly interested in those with clinical and matched control cohorts as well as any longitudinal data to see how stable model representations are over time. Some of the held-out data used here come from different tasks and sometimes separate years of scanning, so we are optimistic regarding this topic.

Finally, we chose functional connectome fingerprinting as a supervised pretraining task, because of its demonstrated utility in different areas of neuroscience (Greene et al., 2018) and because a similar approach has some clinical utility using speech (Moro-Velazquez et al., 2020; Pappagari et al., 2020). However, this is not necessarily an optimal pretraining objective, and different downstream tasks may require different pretraining methods. Brain-age prediction, for example, is another supervised task using labels present in most public data that may distill a representation of brain function that is clinically useful (Leonardsen et al., 2022). Unsupervised methods have also proven to be powerful for leveraging even more pretraining data in text (Devlin et al., 2018) and speech (Chen et al., 2022) processing tasks. These methods could be very powerful if applied to neuroimaging data.

### Conclusion

This work pretrained a flexible generalizable representation for fMRI data. Using connectivity matrices as input allowed us to aggregate over many scanning protocols and to scale up a critical neuroscience task (connectome fingerprinting) to support a new data-driven neural network resource. Along the way we obtain strong performance for connectome fingerprinting. An important implication of these results is that large-scale fingerprint pretraining appears to learn a representation strongly optimized for individual discriminability while only partially capturing broader behavioral and phenotypic structure. This distinction may help guide future work on alternative pretraining objectives better aligned with downstream cognitive or clinical prediction. We hope these results stimulate the field to think of how to flexibly repurpose machine learning representations for new data and tasks and lead to new research on data and neural network-driven biomarkers for neuroscience.

## Data Availability

All data used in this report are publicly accessible via https://openneuro.org, https://db.humanconnectome.org, https://fcon_1000.projects.nitrc.org and https://central.xnat.org. The preprocessing tools used in this report are available from https://fmriprep.org and https://nilearn.github.io. Pretrained model weights along with demo preprocessing and inference scripts are available in Extended Data 2. Code and pretrained model weights are also available at https://doi.org/10.17605/OSF.IO/WFDKX.

10.1523/ENEURO.0370-25.2026.d1 Data 1 Pre-processing boilerplate language generated by fMRIPrep for representative scanning sessions from each dataset. Download Data 1, DOCX file.

10.1523/ENEURO.0370-25.2026.d2 Data 2 Scripts to generate model embeddings with links to access brain2vec model weights. Download Data 2, ZIP file.

## Synthesis

Reviewing Editor: Adrien Peyrache, McGill University Decisions are customarily a result of the Reviewing Editor and the peer reviewers coming together and discussing their recommendations until a consensus is reached. When revisions are invited, a fact-based synthesis statement explaining their decision and outlining what is needed to prepare a revision will be listed below. The following reviewer(s) agreed to reveal their identity: Sarah Yip.

The authors have addressed all the concerns. The manuscript is now suitable for publication.
